# Impact of an eddy dipole of the Mozambique channel on mesopelagic organisms, highlighted by multifrequency backscatter classification

**DOI:** 10.1371/journal.pone.0309840

**Published:** 2024-09-11

**Authors:** Pavanee Annasawmy, Gildas Roudaut, Anne Lebourges Dhaussy

**Affiliations:** CNRS, IRD, Ifremer, LEMAR, Univ Brest, Plouzané, Brest, France; National Research Council, ITALY

## Abstract

The impact of a cyclonic (C), an anticyclonic (AC) eddy and transition zone (TZ), which is the area between the two eddies, on acoustic groups representing various mesopelagic organisms, was investigated using a semi-supervised multifrequency classification approach (hereafter, Escore algorithm). The Escore algorithm involved selecting regions of interest (ROIs) within multifrequency (18, 38, 70, and 120 kHz) echograms and classifying into four clusters or echo-classes using S_v_ differences (S_v18-38_, S_v70-38_, and S_v120-38_). Acoustic densities and diel vertical migration strength varied between the AC, C, and TZ according to the frequency. The vertical stratification of temperature, salinity and fluorescence within the oceanographic structures had varied influences on the vertical structure of each echo-class which represent zooplankton-like organisms, small and large fish with swimbladders, and small and large siphonophores with pneumatophores. The echo-classes within the C were influenced by surface fluorescence, whereas in the AC and TZ, the echo-classes were influenced by deeper fluorescence and strong EKE. Our study provides new insights into the environmental variables within mesoscale and sub-mesoscale features impacting different groups of mesopelagic communities in the Indian Ocean.

## 1. Introduction

The mesopelagic region, 200–1000 m depth [1, 2], is home to gelatinous organisms, zooplankton, and micronekton (crustaceans, mesopelagic fishes and cephalopods of 2 to 20 cm in size which are able to swim independently of ocean currents) [3]. Micronekton is a key trophic link between zooplankton and top predators such as billfishes and tunas [4, 5], and like zooplankton, it plays an important role in the oceanic biological pump through the export of organic carbon [6]. Despite the ecological importance of zooplankton and micronekton, major knowledge gaps exist about their ecological patterns, diversity and taxonomy, especially in the Indian Ocean which is poorly studied. Micronekton is traditionally sampled with mesopelagic trawl nets and active acoustics, both methods presenting sampling limitations. Acoustics give high resolution spatio-temporal series of the horizontal and vertical distributions of organisms inhabiting the mesopelagic zone, unlike trawls which only provide taxonomic descriptions of micronekton at specific sites and time periods. However, it remains difficult to determine the taxonomic groups responsible for the observed backscatter from acoustics.

As an effort to identify major mesopelagic acoustic scatterers, multifrequency approaches have been developed [7–11]. These approaches compare and quantify the difference of mean volume backscattering strength between different frequencies [10] to improve acoustic species identification. While a significant number of backscatter classification techniques has been developed, any particular approach is situation dependent with the classification success depending on the homogeneity of the environment, and relative abundance and frequency response [8]. Most of the former approaches are based on visual observations of the echograms and trawling through specific mesopelagic layers to relate the acoustic backscatter measurements to known organisms [8, 10]. However, during multi-disciplinary cruises, trawling stations may be sporadic and the trawl contents are taxonomically heterogeneous. There is a need to develop multi-frequency classification approaches, especially in the case of sparse sampling coverage and taxonomically heterogeneous trawl stations. The Escore (Ellipsoid score) multifrequency backscatter classification algorithm developed in this study, is inspired from the Z-score classification method [8]. This approach exploits the strengths and follows steps inspired from a wide range of previously described and validated multifrequency backscatter approaches [12]. It uses a semi-supervised selection approach (similarly to [13, 14] but adapted for acoustic layers instead of schools), commonly used clustering techniques on pairwise differences, the Z-score method, multivariate ordination in reduced space, and theoretical scattering models for validation. Previous methods were developed and mostly applied for fish schools or krill aggregations in homogeneous oceanographic conditions. We aimed to adapt the previously described methods to widely different and variable oceanographic settings and on layers of organisms which do not necessarily form schools.

The Mozambique Channel, located between the Southeast African countries of Mozambique and Madagascar island, is highly dynamic due to the presence of mesoscale cyclonic and anticyclonic eddies and sub-mesoscale features such as ocean fronts [15–17]. Mesoscale eddies are rotating bodies of water with horizontal scales of 10–100 km [18, 19]. The eddy dynamics in the Mozambique Channel partly originates from the southern branch of the East Madagascar current being constrained by the bathymetry and forming eddies at approximately 17°S [20]. Eddy dipoles (pair of counter-rotating eddies) which are frequently observed [15, 21], induce the most intense form of upwelling in the Mozambique Channel [22]. Frontal zones between eddies concentrate organic matter leading to phytoplankton enhancement [15]. Previous studies found that cyclonic eddies of the South West Indian Ocean had higher chlorophyll concentrations, zooplankton and micronekton acoustic densities compared to anticyclonic eddies [15, 23, 24]. While studies have shown the impact of eddies and fronts on the vertical distribution of micronekton [24–26], their influence on different mesopelagic groups remains to be investigated.

The main objectives of this study were to implement a multi-frequency acoustic backscatter classification approach to categorize different acoustic groups, and study the influence of environmental processes generated by the presence of a cyclonic (C), an anticyclonic (AC) eddy and transition zone (TZ) on the horizontal and vertical distributions of mesopelagic acoustic densities and the defined acoustic groups.

## 2. Materials and methods

### 2.1 Study site

**Fig 1:**
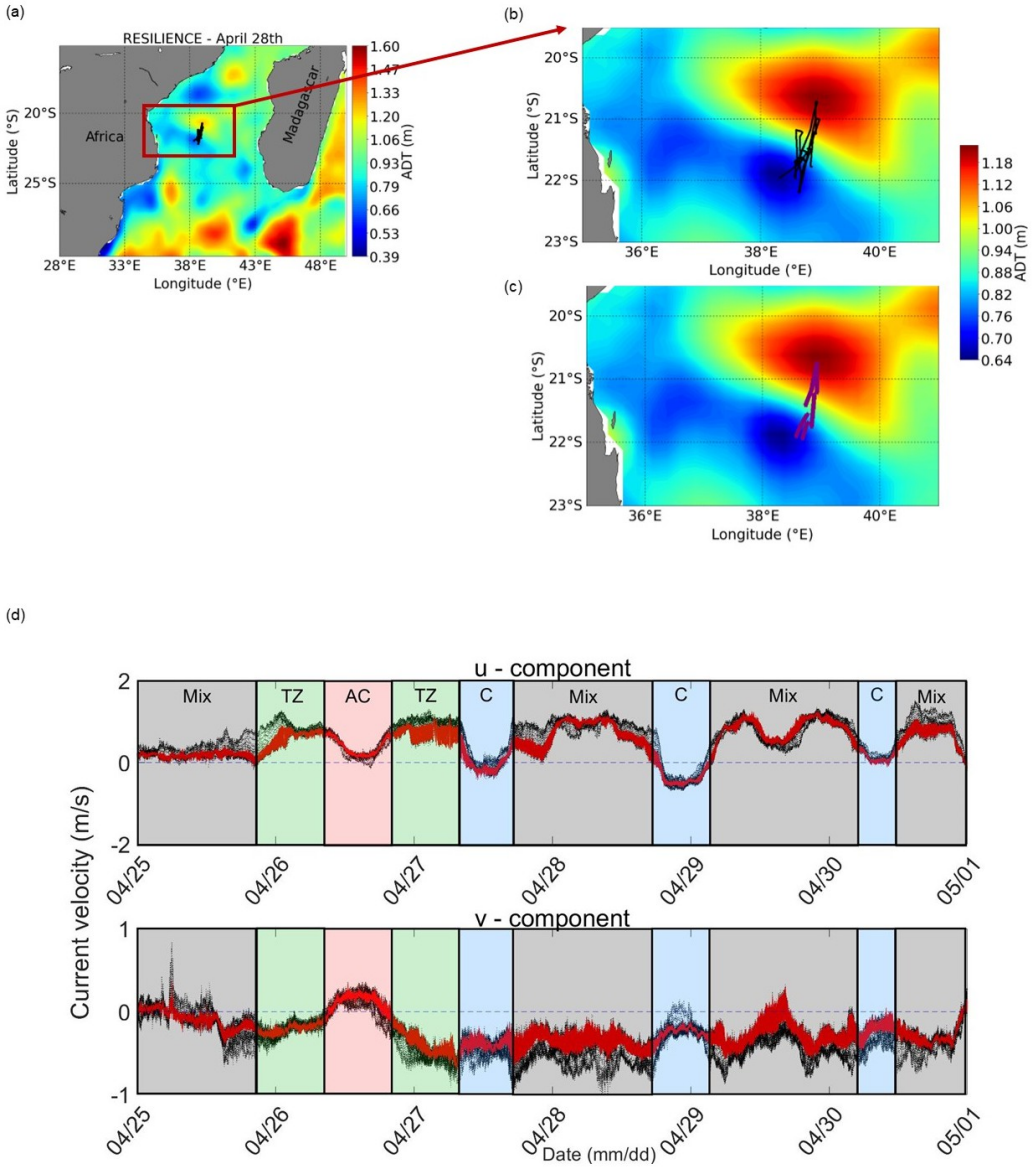
(a) Satellite surface absolute dynamic height (ADT, m) on April 28 within the cyclonic (blue) and anticyclonic (red) eddies. The African and Madagascar Island landmasses are shown in grey. (b) The ship’s track is shown by black lines, and (c) the moving vessel profiler locations are shown by purple lines and are superimposed on the mesoscale eddy field (d) Mean current velocity (m s^-1^) for the u and v velocity components (red data points) and all data points (black) during Leg 1 of the RESILIENCE cruise in the transition zone (TZ), anticyclone (AC) and cyclone (C). A mix zone was determined and discarded from further analyses.

The RESILIENCE cruise was conducted onboard the RV *Marion Dufresne* from April 19 through May 22, 2022 in the South West Indian Ocean (cruise 10.17600/18001917). Permission was granted from the “Réserve Naturelle Nationale des Terres Australes et Antarctiques Françaises” (French Southern and Antarctic Lands) for access to the field site. The cruise was divided into two legs. Only the Leg 1 acoustic dataset from April 25^th^ to 30^th^, was analysed in this study. Environmental and biological datasets from the first leg, which focused on the AC, C, and TZ, will be investigated in more detail (Fig 1a–1c).

### 2.2 Satellite observations

The mesoscale eddy field was described using near real-time L4 Absolute Dynamic Topography (ADT) at a daily temporal resolution and with 0.25° (~ 25 km) spatial resolution. Near real-time ADT is produced and distributed by the E.U. Copernicus Marine Service Information (https://data.marine.copernicus.eu/product/SEALEVEL_GLO_PHY_L4_NRT_008_046/description).

### 2.3 Environmental data

A moving vessel profiler (MVP) undulated between the sea surface and 300 m depth along the ship’s side during 12 transects (12 profiles in total) of 5 hours each, and at a ship speed of 6 knots. The MVP was equipped with conductivity, temperature (Type: 7, AML uCTD—Serial Number: 9113) and fluorescence (ECOFLNTU Fluorimeter / Turbidimeter) sensors. A total of three faulty profiles were discarded. Since the MVP moved slower during ascent, generating more accurate data, only the ascending part of each MVP cycle was used in data analyses. The vertical median binning of the MVP data was conducted at 1 dbar resolution with ~ 25 samples in average per bin and a minimum of 6 samples. The horizontal interpolation was conducted at 1-min sampling resolution.

A ship-mounted thermosalinograph continuously recorded sea surface temperature and salinity along the vessel’s track within 5 m of the sea surface. A CTD (Conductivity Temperature Depth) rosette system was equipped with a SBE911+ probe to measure temperature and salinity down to 1000 m. Underway current profiles were measured along the ship’s track with a 150 kHz RDI Ocean Surveyor II Acoustic Doppler Current Profiler (S-ADCP) using a time average of approximately 1 min and depth bins of 16 m from the surface to approximately 400 m. The S-ADCP report velocities in the north (v component) and east (u component) directions. Current data were processed using the CASCADE software [27] which allows flagging and filtering. A tidal correction was applied to the S-ADCP dataset before aggregating measurements to 8-m depth bins. The eddy kinetic energy (EKE, m^2${\text{s}}^{-2}=\frac{1}{2}(u^{2}+v^{2})$^, which is a proxy for mesoscale dynamics in the ocean, was calculated from the u and v velocity components.

### 2.4 Classification of acoustic and environmental data

Acoustic and environmental data points were divided into three categories based on their location relative to the core and edges of the AC and C, and the TZ. This classification (Table 1) was based on current data from the S-ADCP, hydrological data (temperature and salinity gradients recorded by the thermosalinograph) and fluorescence gradients measured by the MVP. Altimetry data (ADT) at daily near real-time resolution were used to visualize the progression of the mesoscale features. Sharp gradients in the environmental variables (S1 File) and the v component from the S-ADCP were used to classify the acoustic and environmental data points according to their location with respect to the AC, C and TZ. At the beginning of Leg 1, the AC was headed north (v > 0 and u ~ 0) and the C was headed south (v < 0 and u~ 0) (Fig 1d). The TZ was characterized by very strong currents towards the east (u > 0). The “mix” zone (Fig 1d) corresponded to sampling alternatively between the AC and TZ such that it was not possible to divide this zone into AC or TZ, and was hence discarded from further analysis.

**Table 1:** Classification of Leg 1 acoustic transect based on the location of the data points within the cyclonic, anticyclonic eddies, transition and mix zones.

Start date (dd/mm)	Start time (hh:mm:ss)	End date	End time	Structure
25/04	20:33:52	26/04	09:02:54	Transition zone
26/04	09:02:55	26/04	19:00:58	Anticyclone
26/04	19:00:59	27/04	07:41:36	Transition zone
27/04	07:41:37	27/04	17:10:38	Cyclone
27/04	17:10:39	28/04	17:33:52	Mix
28/04	17:33:53	29/04	03:26:07	Cyclone
29/04	03:26:08	30/04	04:24:11	Mix
30/04	04:24:12	30/04	11:22:15	Cyclone
30/04	11:22:16	30/04	23:51:17	Mix

### 2.5 Acoustic data collection and processing

Acoustic data were continuously acquired in narrowband (i.e., continuous wave mode) using a Simrad EK80 echosounder at 5 frequencies: 18, 38, 70, 120, and 200 kHz. The transducers are hull-mounted at a depth of 6 m below the sea surface. The echosounder was calibrated prior to the cruise, in January 2022 at Reunion Island in the Indian Ocean, using a 38.1 mm tungsten carbide calibration sphere (with 6% cobalt binder), following [28]. The Simrad EK80 software was used to obtain echosounder calibration parameters (Table 2) that were used in data processing.

**Table 2:** Echosounder parameter settings used during the acoustic data acquisition and processing.

Frequencies (kHz)	18	38	70	120	200
Maximum power (W)	1600	1000	750	200	90
Pulse duration (ms)	1.024	1.024	1.024	1.024	1.024
Ping interval (s)	3	3	3	3	3
S_a_ correction	-0.02	0.01	-0.04	-0.07	-0.06
Transducer gain (dB)	21.35	26.67	26.93	26.08	25.12
Beamwidth alongship (°)	10.46	7.02	6.55	6.57	6.48
Beamwidth arthwartship (°)	10.46	7.02	6.55	6.57	6.48
Angle offset alongship (°)	0.01	-0.01	0.01	0.02	-0.01
Angle offset arthwartship (°)	0	0	-0.08	0.09	0.05
Absorption Coefficients (dB/km) at Temperature = 26°C and Salinity: 35.5	1.51	6.53	20.2	47.9	88.7
Depth range (m)	1500	800	450	250	120

The acoustic data were visualized and processed using Matecho software (v. 7, 20220511), an open source IRD tool computed with Matlab 7.11.0.184, and based on Ifremer’s Movies3D software [29]. The top 15 m of the water column was removed from analysis at all frequencies to account for the acoustic detection of the surface turbulence. Background, transient and impulsive noises, and attenuated signals were removed using the algorithms designed in [30, 31], implemented in Matecho. Temperature and salinity vertical profiles obtained from the CTD stations during the survey were used to estimate the sound velocities, absorption coefficients, and to correct S_v_ estimates. Matecho evaluates twilight periods (night, sunrise, day, sunset) from the ping time and the geographical position using a MATLAB “Suncycle” script [29]. Acoustic data were echo-integrated in s_A_ (m^2^ nmi^-2^) and s_v_ which was log-transformed to S_v_ in Matecho at an elementary sampling distance unit (ESDU) of 1.5 m vertically and 3 pings horizontally, and a threshold of −100 dB re 1 m^-1^ (hereafter dB). The volume backscattering strength, S_v_, represents mesopelagic acoustic densities.

### 2.6 Multifrequency acoustic backscatter classification

**Fig 2:**
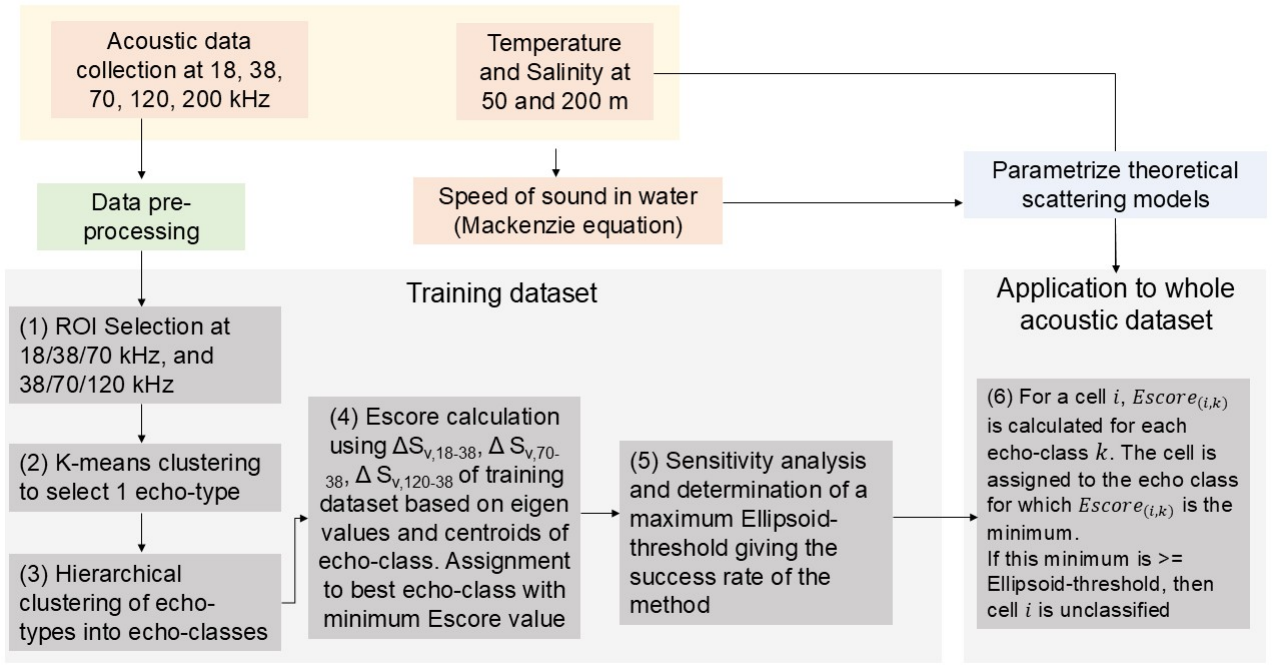
Flowchart summarizing the Escore methodology introduced in this study.

The Escore algorithm relies on dB differences between pairs of frequencies by subtracting the backscatter measurements of each available frequency relative to the 38 kHz. The code used to implement the Escore algorithm is publicly available through IRD Forge (https://forge.ird.fr/lemar/active_acoustics/multifrequency_classification/-/tree/master/Escore). The Escore algorithm was applied to classify the multifrequency acoustic backscatter into echo-classes (Table 3) through the stepwise procedures summarized below and in Fig 2.

**Table 3:** Glossary of terms used in the acoustic backscatter classification approach.

Terms	Definitions
Region of Interest (ROI)	Manually chosen rectangular section of the RGB echogram which contains a specific structure of interest with consistent frequency responses.
Echo-type	Each echo-type encompasses a set of individual pixels classified into the same cluster from the ROI definition and the K-means clustering (step 2).
Library	The whole set of echo-types identified from the training dataset.
Echo-class	Each echo-class encompasses several echo-types with similar characteristics and is the result of the echo-type classification (i.e., hierarchical classification) of a library (step 3). The echo-classes form the learning/reference database for the classification of the whole acoustic data.

The following steps are a brief summary and the full methodology is provided in S2 File:

Step (1): Due to the presence of layers of organisms (instead of discrete schools) on our echograms, a semi-supervised approach consisting of manually selecting Regions of interest (ROI) from Red Green Blue (RGB) composite echograms [11] at 18/38/70 kHz (n = 77 ROI) and 38/70/120 kHz (n = 138) frequencies was implemented. For the 18/38/70 kHz RGB composite echograms, the S_v_ values of the 18 kHz frequency was color-coded in red; 38 kHz in green; and 70 kHz in blue. For the 38/70/120 kHz composite echogram, the 38 kHz frequency was color-coded in red; 70 kHz in green; and 120 kHz in blue (S1 Fig in S2 File). The ROI were chosen based on visible structures having a homogeneous frequency response in a layer with consistent acoustic and spatial characteristics. The ROI may contain different biological structures or patches that will be discriminated in the next step. Due to use of the 120 kHz frequency, the ROI were chosen only within the first 250 m of the water column.

Step (2): The pixels within each ROI were subjected to a K-means clustering based on the relative frequency responses at 18, 70, and 120 kHz [32] in dB (ΔS_v,18−38_, ΔS_v,70−38_, and ΔS_v,120−38_) to retain one cluster representing a single coherent visible acoustic structure, i.e., one echo-type (Table 3; S2 Fig in S2 File). This step is conducted to allow the selection of only 1 acoustic structure with limited variability in frequency responses before conducting a hierarchical clustering and ordination. These echo-types constituted the training dataset before applying the Escore algorithm to the whole acoustic dataset.

Step (3): Three mean S_v_ differences (ΔS_v,18−38_, ΔS_v,70−38_, and ΔS_v,120−38_) were calculated from the 215 echo-types and classified into four echo-classes using hierarchical cluster analysis which is fully described in S3 Fig in S2 File. The optimum number of echo-classes was determined using the NbClust package in R [33]. Out of 20 indices implemented in NbClust, 11 selected four optimal number of echo-classes. Cluster performance was tested using a random forest algorithm as described in S2 File. The classification error was estimated from a random subset of the bootstrap data, and an error rate was returned for each echo-class. The echo-types were robustly classified into four clusters/echo-classes at 99.4%.

Step (4): A three-dimensional ordination plot was generated to visualize the four echo-classes along the S_v_ difference axes between S_v18-38_ (x-axis), S_v70-38_ (y-axis) and S_v120-38_ (z-axis) (S4a Fig in S2 File). Data points representing the S_v_ differences of each echo-class were plotted around a centroid of an ellipsoid at mean ± 2 standard deviations. Relative frequency response curves of each echo-class were drawn so as to assign the frequency with dominant backscatter to each echo-class. Echo-classes 1, 2, and 4 were assigned to the dominant 38 kHz frequency, and echo-class 3 to the 18 kHz frequency based on S4b Fig in S2 File. To differentiate echo-classes 1, 2, and 4, all having 38 kHz dominant frequencies, the Escore algorithm was computed in the following steps.

Step (5): The Escore is a sum of squared independent normal random variables which follow a chi-square distribution with three degrees of freedom. For an echo-integrated cell *i* characterized by 3 relative frequency differences relative to the 38 kHz, *Escore*_*(i*,*k)*_ is calculated for each echo-class *k*. The cell is assigned to the echo-class for which *Escore*_*(i*,*k)*_ is the minimum. If this minimum is > = Ellipsoid-threshold, then cell *i* is unclassified.

Sensitivity tests were conducted on the training dataset. This allows determination of the percentage echo-integrated cells that were well classified into each echo-class, mis-classified and not classified. An Ellipsoid-threshold value of 25 appeared to best classify each data point into one of the four echo-classes while limiting the percentage echo-integration cells and s_A_ mis-classified and decreasing those which were not classified (S5 Fig in S2 File).

Step (6): The Escore calculation (fully described in S2 File) was run on the whole acoustic echo-integration cells (including the cells from the training dataset) to classify each data point of the chosen leg into one of the four echo-classes (S6 Fig in S2 File).

Echograms were generated for each echo-class from 15 to 250 m depth, and were used in further statistical analyses (Section 2.8).

### 2.7 Validation of the Escore algorithm

The low number of trawls, all conducted within the TZ during the first leg, could not be used to validate the Escore algorithm. Potential candidates of each echo-class were investigated by classifying theoretical organisms, whose TS(f) was calculated using previously published and validated theoretical scattering models. The scattering models used to investigate the biological significance of each echo-class defined from Section 2.6 were the following: Distorted Wave Born Approximation (DWBA) with parameters for copepods and euphausiids [34, 35]; randomly oriented fluid bent cylinder with parameters for shrimp and salp [36]; high-pass dense fluid sphere with parameters for gastropods [36]; hybrid model with parameters for pneumatophores of siphonophores and small gaseous swimbladdered fish [37]; model for gas bubble alone [38]. Each scattering model was parametrized using values of speed of sound in the water calculated from temperature and salinity at 25 m, and 200 m depths using the Mackenzie equation, density contrast (for lack of other measures), orientation and the length-to-width ratio of organisms as determined previously by [35–37] (Refer to S3 File for parameters used in the scattering models). For each scattering model, frequency responses were calculated for a set of size intervals at 18, 38, 70, and 120 kHz, and the S_v_ (for 1 organism m^-3^) frequency differences (ΔS_v,18−38_, ΔS_v,70−38_, and ΔS_v,120−38_) were calculated prior to classifying the model into one of the four echo-classes at a maximum Ellipsoid-threshold of 25.

### 2.8 Statistical analyses

#### 2.8.1 Environmental variables

The differences in fluorescence, salinity and temperature values between the AC, C and TZ in the first 100 m, between 100–200 m and 200–300 m, were investigated using Kruskal-Wallis (KW) tests and pairwise comparisons.

#### 2.8.2 Acoustic metrics

The following acoustic metrics were calculated following [39]:

$$Total\;s_{A}=\sum s_{A-i},$$ (1)

where *s*_*A-i*_ is the daytime or nighttime area backscattter for a given 1.5-m depth bin between the surface and the maximum echo-integrated depth for each frequency (18 kHz: 1000 m; 38 kHz: 800 m; 70 kHz: 500 m; 120 kHz: 250 m; 200 kHz: 120 m).

$$DVM\;\left(diel\;vertical\;migration\right)\;strength=1-\left(\frac{s_{A-meso-night}}{s_{A-meso-day}}\right),$$ (2)

where the DVM strength > 0, *s*_*A-meso-night*_ and *s*_*A-meso-day*_ are the integrated daytime or nighttime area backscatter in the mesopelagic realm, i.e., from 200 m to the maximum echo-integrated depth for the 18, 38 and 70 kHz frequencies (referred to as “meso” in Eq 2).

#### 2.8.3 Acoustic densities between oceanographic structures, frequencies and depth categories

The day and night mean and standard deviations of volume backscattering strength (MacLennan et al., 2002), S_v_, were calculated for the AC, C and TZ, and plotted. To investigate the difference in NASC (Nautical Area Scattering Coefficient or s_A_) between the AC, C and TZ for the 18, 38 and 70 kHz frequencies, the water column was separated into three depth categories: 15–200 m (surface), 200–400 m (intermediate) and below 400 m (deep), with diurnal and nocturnal periods being separately analysed using KW tests and pairwise comparisons. Only surface and intermediate depth categories were identified for the 120 kHz, and only a surface layer for the 200 kHz (due to the sampling range limitations of both frequencies), and investigated using KW tests.

#### 2.8.4 Influence of the environment on the acoustic densities of echo-classes

PERMANOVA were conducted using the “adonis2” function in R [40] to test whether there was a difference in NASC of the echo-classes (s_A_ at each dominant frequency) between the oceanographic structures (AC, C, and TZ), and time of day (day and night). To investigate the relative importance of candidate covariates influencing mesopelagic organisms, acoustic data from each echo-class and selected environmental variables from the MVP and S-ADCP were matched spatially and temporally, before constructing generalized additive mixed models (GAMMs) using the R package mgcv (v 1.8.41) [41]. See S4 and S5 Files for full GAMM fitting and specification. Since regularly spaced acoustic data are likely to exhibit a degree of spatial autocorrelation, resulting in violation of the assumption of independence between samples, we nested an autoregressive correlation structure of order 1 (corAR1). Similar modelling approaches have been used to investigate relationships between acoustic and environmental data [e.g., 42–44].

GAMMs allowed the identification of the environmental variables best influencing s_A_ of each echo-class, but do not provide information related to the oceanographic structures. To explore the relationships between environmental variables in the AC, C and TZ, we conducted Canonical Analyses of Principal Coordinates (CAP) on Euclidean distance matrices of the acoustic data of each echo-class using the R vegan package (v. 2.6.4), similar to [39]. CAP analysis is a constrained ordination which aims to find axes in the predictor space that best explain variation in the multivariate response [45]. For the CAP analyses, we used the significant environmental variables identified from the GAMMs (See section 3.3.1). The result is represented as a two-dimensional graph, with the environmental variables plotted as centred vectors. The magnitude of variation of each environmental variable is proportional to the length of the vector, the direction of which indicates increasing values of the variable.

## 3. Results

### 3.1 Prevailing environmental conditions at the study site

**Fig 3:**
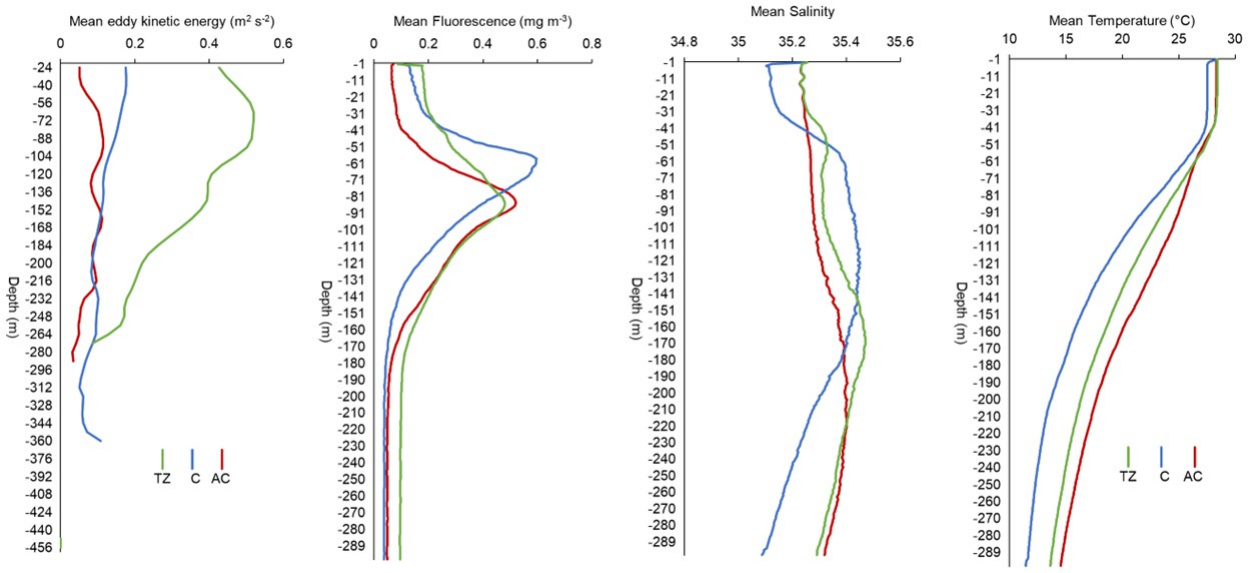
Vertical profiles of mean eddy kinetic energy (m^2^ s^-2^), fluorescence (mg m^-3^), salinity and temperature (°C) within the transition zone (TZ), cyclone (C), and anticyclone (AC).

The sampling effort was focused within the AC, C and TZ during the first leg of the RESILIENCE cruise. Sharp gradients were observed in the temperature and salinity profiles derived from the thermosalinograph, indicating the shift from the TZ to the C (S1 File). A peak in the mean EKE was observed at 64 m in the TZ (0.52 m^2^ s^-2^) before decreasing to a minimum of 0.09 m^2^ s^-2^ at 272 m. Within the C, mean EKE showed a decreasing trend from 0.18 m^2^ s^-2^ at 24 m to 0.09 m^2^ s^-2^ at 216 m, before increasing to 0.11 m^2^ s^-2^ at 360 m (Fig 3). Mean EKE within the AC showed an increasing trend from 0.05 m^2^ s^-2^ at 24 m to 0.11 m^2^ s^-2^ at 160 m, before showing a decreasing trend.

Fluorescence and salinity were significantly different between the AC, C and TZ between the surface and 300 m (KW, pairwise comparisons, p < 0.05). A shallow peak in fluorescence values was seen in the C (0.59 mg m^-3^ at 60 m) which was also saltier (mean ± standard deviation: 35.42 ± 0.02) compared to the AC (fluorescence: 0.52 mg m^-3^ at 84 m; salinity: 35.29 ± 0.02) and TZ (0.48 mg m^-3^ at 85 m; 35.34 ± 0.04) between 53 and 141 m (Fig 3). The AC was saltier than the C between 200–300 m (C: 35.20 ± 0.045; AC: 35.37 ± 0.007). The temperature profiles showed similar vertical patterns in the AC, C and TZ, with a general decrease in temperature with depth. The C was colder (mean ± standard deviation: 18.2 ± 0.72°C) than the AC (21.3 ± 0.17°C) (KW, pairwise comparisons, p < 0.05) from the surface to 300 m. In the first 69 m, the AC and TZ showed similar temperature values (AC: 27.0 ± 0.19°C; TZ: 26.6 ± 0.88°C) (KW, pairwise comparisons, p > 0.05), but the TZ was colder than the AC by 0.4 to 2°C below that depth (Fig 3).

### 3.2 Comparison of acoustic densities and metrics between the AC, C and TZ

**Fig 4:**
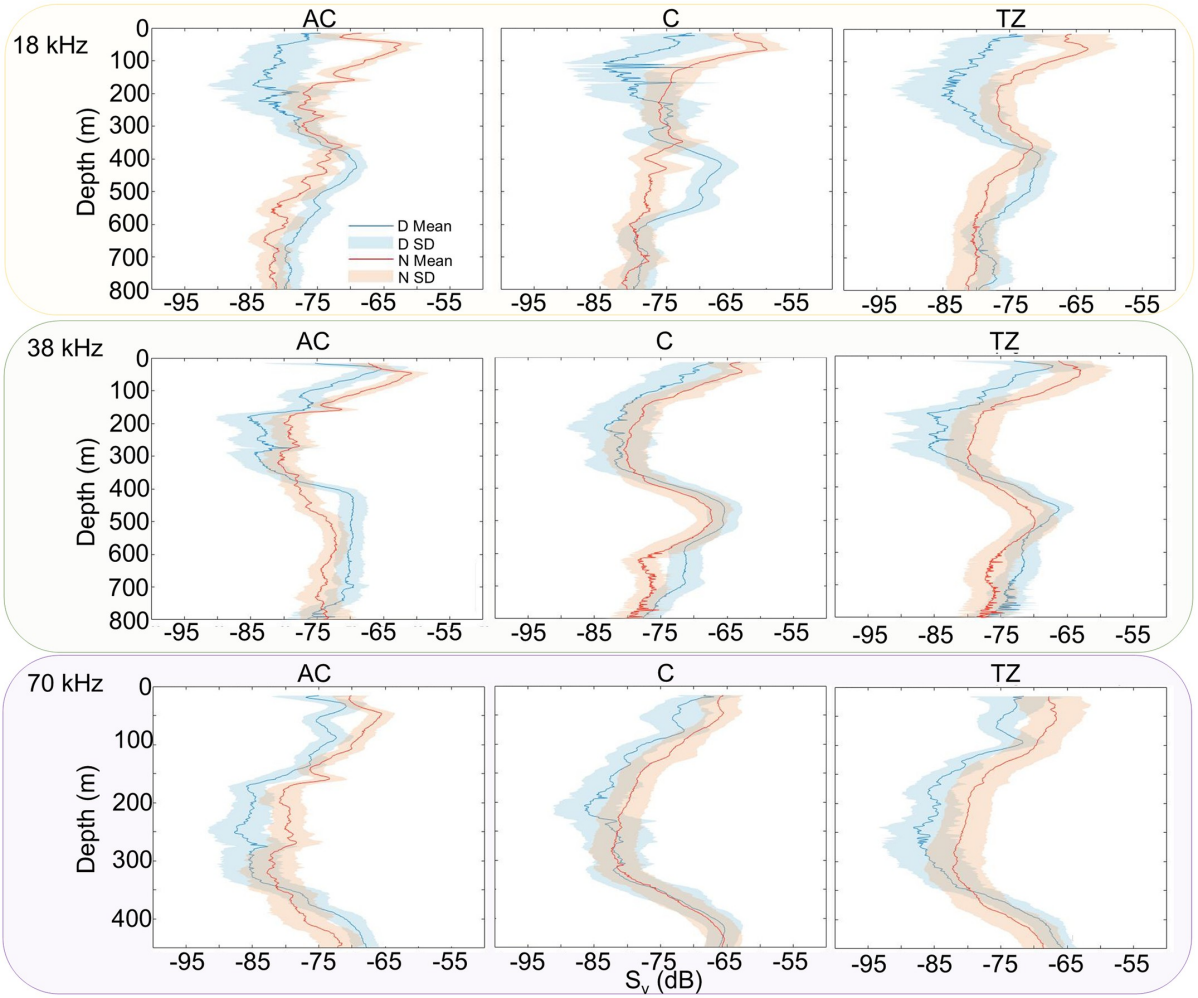
Day and night mean vertical S_v_ (dB) profiles ± standard deviations (SD) within the anticyclone (AC), cyclone (C) and transition zone (TZ) at the 18, 38, and 70 kHz frequencies.

Vertical profiles of mean S_v_ varied between the 18, 38 and 70 kHz frequencies with depth (Fig 4), although similar day and night patterns were observed with higher acoustic densities in the top 200 m at night compared to daytime across the AC, C and TZ. Higher acoustic densities were observed between 400 and 600 m during the day compared to nighttime across the AC, C and TZ at 18 and 38 kHz frequencies.

Acoustic metrics for each frequency and each zone are summarized in Table 4. The mean daytime total NASC was greater in the C compared to the AC and TZ at the 18, 38, and 70 kHz, whereas it was greater in the AC compared to the C and TZ at the 120 and 200 kHz. During nighttime, the total NASC was greater in the C than the AC and TZ at the 18, 70, 120 and 200 kHz. At the 38 kHz frequency, the total nighttime NASC values showed little variability between the AC, C, and TZ. The proportion of mesopelagic communities that appeared to migrate (i.e., the DVM strength) was greater in the C than the AC and TZ at the 18 kHz, but lower at the 70 kHz. At the 38 kHz, the DVM strength was greater in the AC compared to the C and TZ.

**Table 4:** Mean ± standard deviation daytime (D) and nighttime (N) 18–200 kHz acoustic metrics in the anticyclone (AC), cyclone (C), and transition zone (TZ). The maximum echo-integrated depths were 1000 m, 800 m, 450 m, 250 m, and 120 m for the 18, 38, 70, 120 and 200 kHz, respectively.

Frequencies (kHz)	Time of day	Oceanographic structures	Total s_A_ (m^2^ nmi^−2^)	DVM Strength
18	D	AC	1022 ± 250	0.38 ± 0.63
		C	1565 ± 450	0.61 ± 0.73
		TZ	940 ± 156	0.29 ± 0.19
	N	AC	2008 ± 168	
		C	2688 ± 652	
		TZ	1903 ± 686	
38	D	AC	2139 ± 335	0.50 ± 0.64
		C	2518 ± 611	0.42 ± 0.45
		TZ	1907 ± 414	0.44 ± 0.06
	N	AC	2583 ± 268	
		C	2495 ± 805	
		TZ	2435 ± 812	
70	D	AC	593 ± 117	0.51 ± 0.38
		C	1034 ± 341	0.05 ± 0.08
		TZ	731 ± 231	0.46 ± 0.20
	N	AC	921 ± 96	
		C	1518 ± 447	
		TZ	1061 ± 360	
120	D	AC	272 + 42	
		C	190 + 125	
		TZ	175 + 45	
	N	AC	603 + 60	
		C	626 + 222	
		TZ	591 + 203	
200	D	AC	293 + 56	
		C	251 + 209	
		TZ	199 + 44	
	N	AC	611 + 70	
		C	768 + 357	
		TZ	638 + 232	

**Fig 5:**
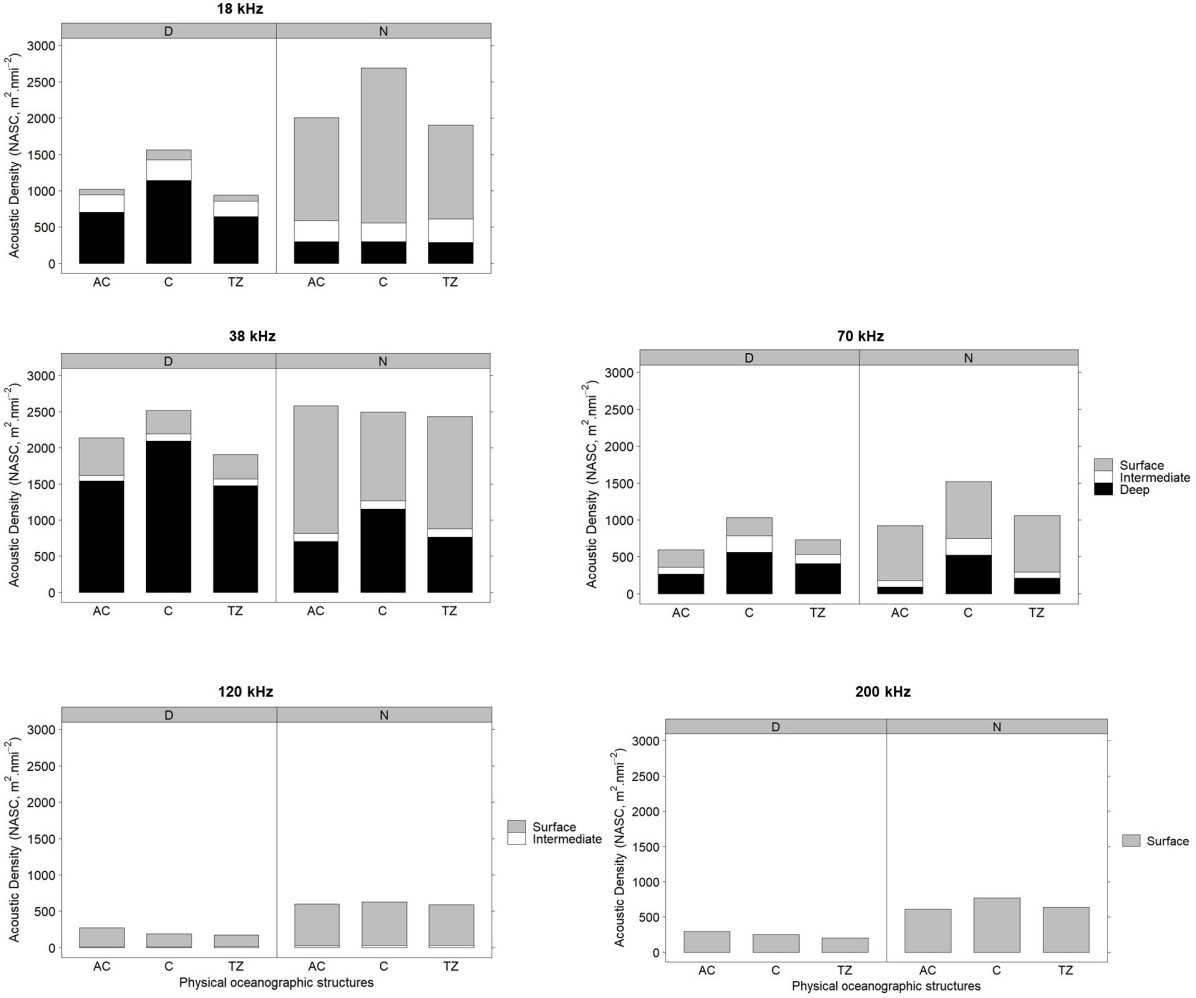
Stacked bar charts of NASC, s_A_, during day (D) and night (N) at the 18, 38, 70, 120, and 200 kHz within the surface (top 200 m), intermediate (200–400 m), and deeper layers (> 400 m) in the anticyclone (AC), cyclone (C), and transition zone (TZ).

In the deep depth category, the C showed higher NASC during the day at the 18, 38, and 70 kHz compared to the AC and TZ (KW, pairwise comparisons, p < 0.05; Fig 5). The influence of the C was also observed at night with higher NASC in the deep layer at the 38 and 70 kHz compared to the AC and TZ (KW, pairwise comparisons, p < 0.05; Fig 5). In the surface layer, the patterns of acoustic densities varied between the AC and C according to the frequency during both day and night. During the day, the C showed higher NASC in the surface layer at the 18 and 70 kHz compared to the AC (KW, pairwise comparisons, p < 0.05; Fig 5). However, at the 38, 120 and 200 kHz, the AC showed higher NASC than the C in the surface layer during the day (KW, pairwise comparisons, p < 0.05). The C showed higher NASC at night in the surface depth category at the 18 and 200 kHz compared to the AC (KW, pairwise comparisons, p < 0.05). However, the AC (1769 m^2^ nmi^-2^) showed higher NASC than the C (1229 m^2^ nmi^-2^) at the 38 kHz frequency (KW, pairwise comparisons, p < 0.05), and the NASC values were similar between the AC, C, and TZ at the 70 kHz frequency (KW, pairwise comparisons, p > 0.05; Fig 5) in the surface layer at night.

### 3.3 Densities of echo-classes

The NASC of echo-classes 1, 3, and 4, were significantly different between the AC, C, and TZ during daytime and nighttime (PERMANOVA, pairwise comparisons, p < 0.05). The mean daytime total NASC of echo-classes 1, 3 and 4 was higher in the C compared to the AC and TZ (Table 5). Echo-class 2 showed higher daytime NASC in the AC compared to the C and TZ. The nighttime NASC of the echo-classes was more variable between oceanographic structures, with the echo-class 1 showing greater values in the TZ compared to the AC and C. Echo-class 2 showed similarly high values in the AC and TZ compared to the C. Echo-class 3 showed higher values in the C compared to the AC and TZ, and echo-class 4 showed higher values in the AC and lower values in the C.

**Table 5:** Mean ± standard deviations daytime (D) and nighttime (N) nautical area scattering coefficient, s_A_ (m^2^ nmi^-2^) from 15 to 250 m, of echo-classes 1 to 4 in the anticyclone (AC), cyclone (C), and transition zone (TZ).

Echo-class number	Time of day	Oceanographic structures	Mean ± standard deviation
Echo-class 1	D	AC	184 ± 48
		C	191 ± 118
		TZ	141 ± 51
	N	AC	75 ± 40
		C	52 ± 59
		TZ	88 ± 83
Echo-class 2	D	AC	372 ± 172
		C	143 ± 120
		TZ	210 ± 178
	N	AC	176 ± 94
		C	23 ± 65
		TZ	177 ± 220
Echo-class 3	D	AC	42 ± 56
		C	142 ± 176
		TZ	62 ± 48
	N	AC	933 ± 223
		C	1970 ± 608
		TZ	890 ± 647
Echo-class 4	D	AC	109 ± 49
		C	182 ± 145
		TZ	104 ± 65
	N	AC	1708 ± 325
		C	769 ± 555
		TZ	1275 ± 640

**Fig 6:**
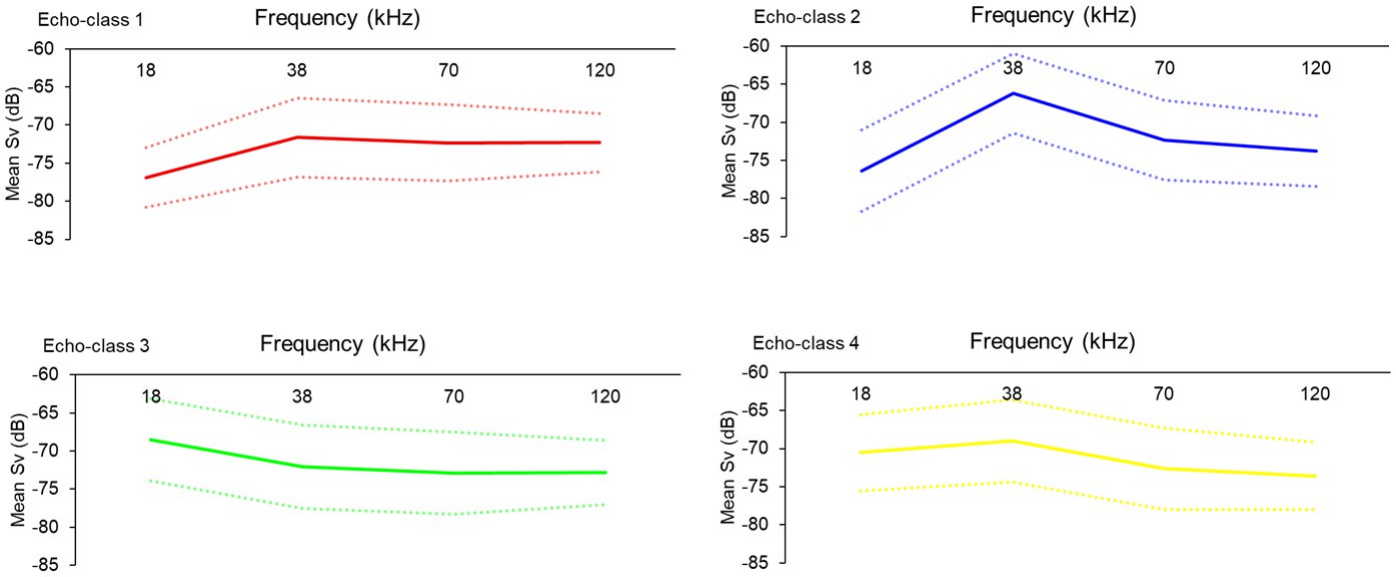
Frequency response curves at 18, 38, 70, and 120 kHz frequencies of echo-classes 1 to 4 during nighttime. Mean S_v_ is shown by the solid lines and confidence intervals with the dashed lines.

The frequency response curves of echo-class 1 peaked at 38 kHz and levels off at higher frequencies (Fig 6). Echo-classes 2 and 4 also peaked at 38 kHz and decreased at 70 kHz. The frequency response curve of echo-class 2 increased with a steeper slope from 18 kHz and decreased with a steeper slope at higher frequencies compared to echo-class 4. Echo-class 3 was dominant at 18 kHz and decreased at higher frequencies.

#### 3.3.1 Environmental influence on echo-classes

**Fig 7:**
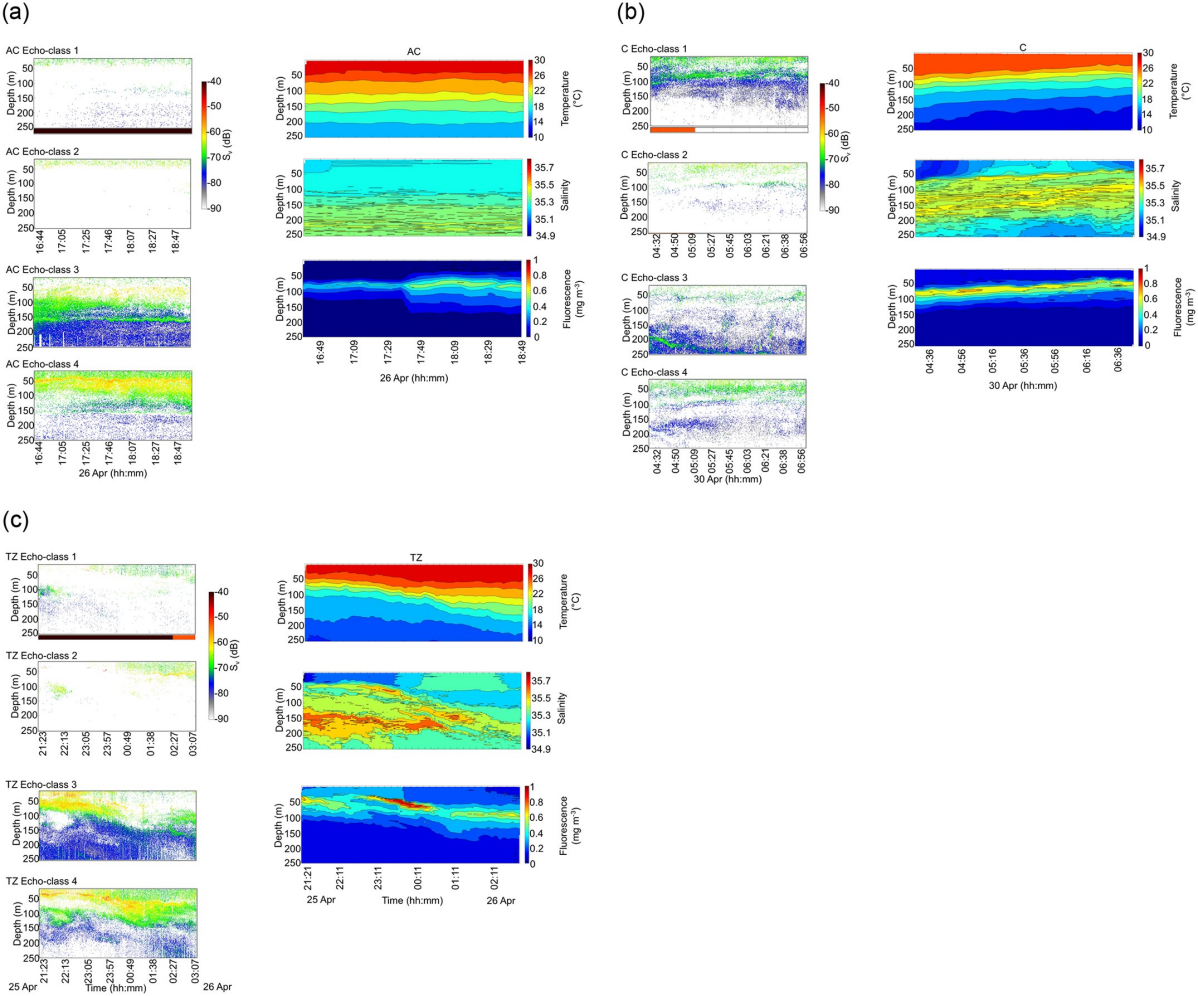
Left-hand panels: Echograms of S_v_ values from the surface to 250 m for the echo-classes 1 to 4 in the (a) anticyclone (AC), (b) cyclone (C) and (c) transition zone (TZ). Nighttime is denoted by the black rectangles below the echograms, sunrise in orange, and daytime in white. Right-hand panels: Vertical section plots of temperature (°C), salinity, and fluorescence (mg m^-3^) in the (a) AC, (b) C and (c) TZ, corresponding spatially and temporally to the RGB echograms of the left-hand panels.

The four echo-classes were vertically structured according to the stratification of the water column in the AC, C and TZ (Fig 7). Echograms showed echo-class 3 being dominant between 100–150 m of the water column during nighttime in the AC, corresponding with moderate temperature, salinity and fluorescence values compared to other depths (Fig 7a). Echo-class 4 was dominant at 50 m, corresponding with the 28°C isotherm, and the maximum fluorescence values extending from 50 to 100 m. In the C, the distribution of echo-class 1 followed the shallowing of the thermocline, halocline and fluorescence maximum depth from 100 m to 50 m during sunrise and daytime (Fig 7b). Echo-classes 2 and 4 were dominant in the first 50 m of the water column in the C where temperature values were high, and salinity and fluorescence low. Echo-class 3 showed a downward migration as the sun rose, from 200 m to deeper depths in the C below the thermocline, halocline and fluorescence maximum depth. In the TZ, echo-classes 1 and 2 did not show a marked vertical stratification with the environmental variables (Fig 7c). Echo-class 3 was highly dominant in the first 50 m during nighttime in the TZ where fluorescence values were highest and deepened with the deepening halocline and fluorescence maximum depth during sunrise. Echo-class 4 peaked in a fine layer complementary to echo-class 3 above the maximum fluorescence values, and showed the highest values in the first 150 m during sunrise, following the temperature, salinity and fluorescence gradients.

**Fig 8:**
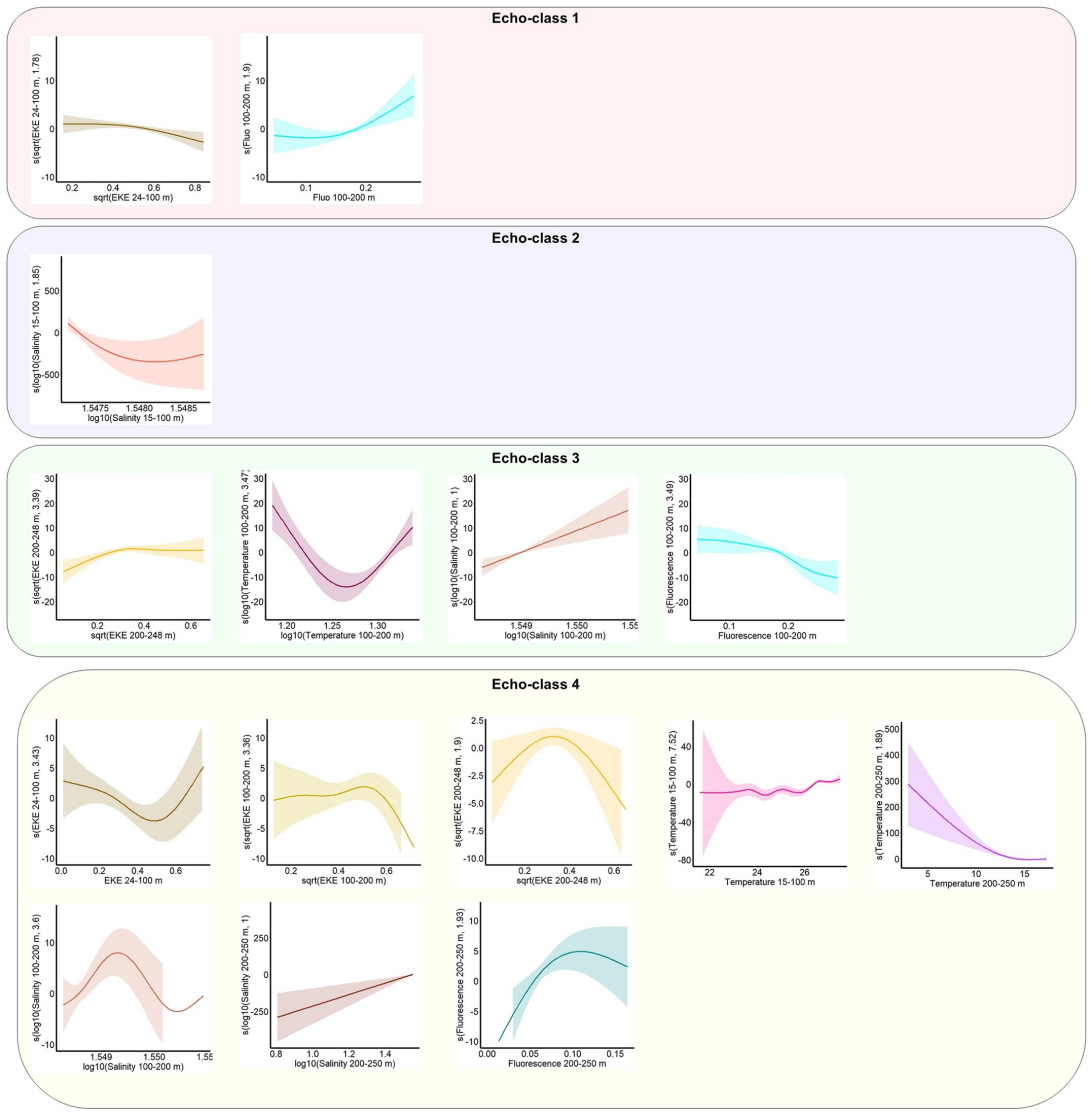
Smooth functions for the GAMMs showing the influence of significant covariates on the NASC values of echo-classes 1 to 4. The y-axes show the smooth function of each covariate, with the estimated degrees of freedom in brackets. The predicted models are shown by solid lines and the 95% confidence intervals by filled contours. Data observations are shown on the x-axis.

The selected GAMMs of echo-classes 1 to 4 explained 59%, 31%, 89%, and 93% of model variances with different oceanographic variables influencing predicted s_A_ values of echo-classes. For echo-class 1, the main variables explaining the variance of s_A_ values were the mean EKE between 24 and 100 m and the fluorescence between 100 and 200 m (p < 0.05) (Fig 8). The only variable explaining the variance of s_A_ values for echo-class 2 was the salinity between 15 and 100 m. The s_A_ values of echo-class 3 were positively influenced by salinity between 100–200 m, negatively by fluorescence 100–200 m, increased due to the mean EKE 200–248 m before reaching a plateau at high EKE values, decreased at low temperatures and increased at higher temperatures (p < 0.05). The s_A_ values of echo-class 4 were influenced by the mean EKE between 24–248 m, fluorescence 200–250 m, salinity 100–250 m, and temperature 15–100 m and 200–250 m (p < 0.05).

**Fig 9:**
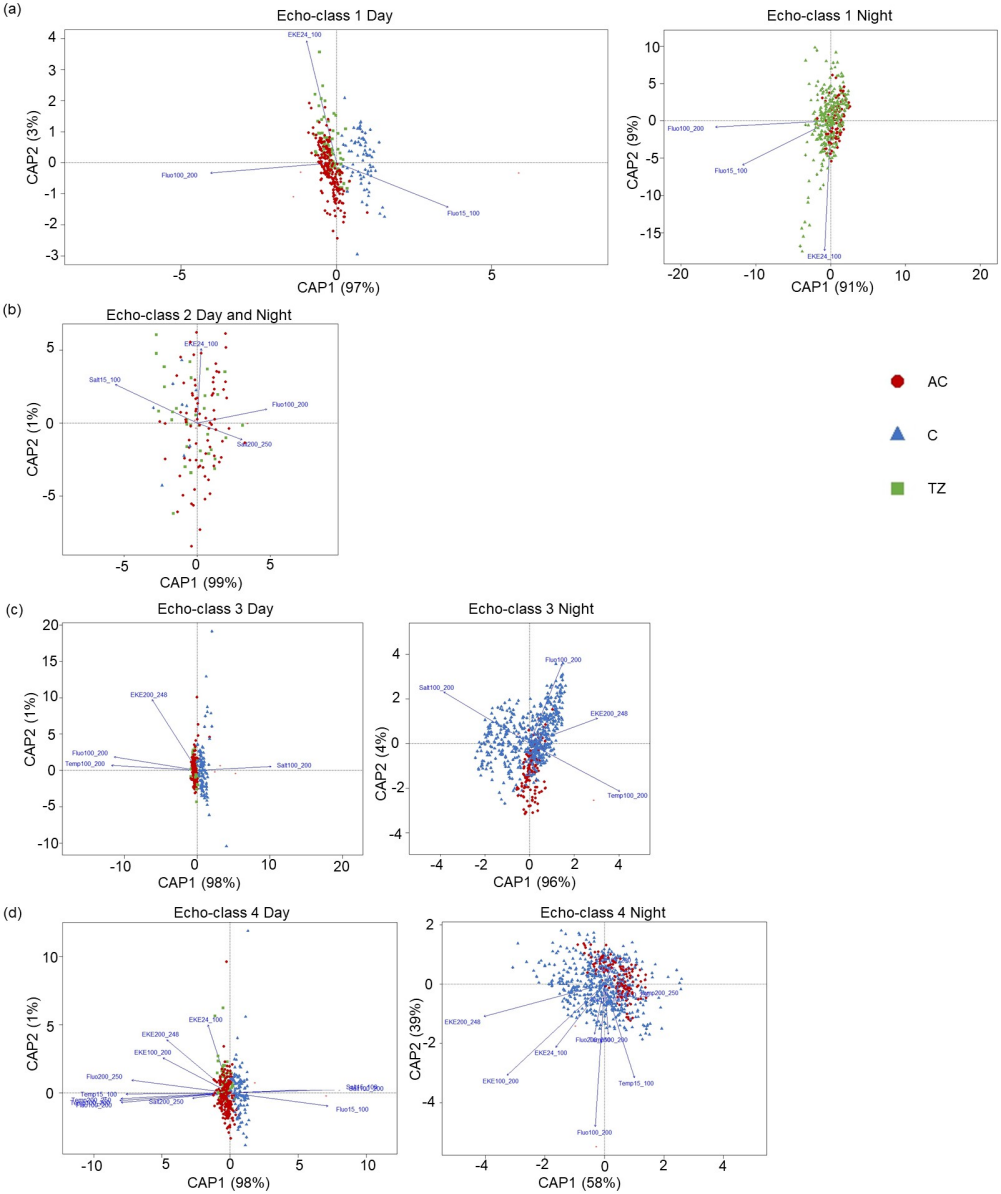
Daytime and nighttime canonical analysis of principal components (CAP) for (a) echo-class 1, (b) echo-class 2, (c) echo-class 3, and (d) echo-class 4. Each point represents one s_A_ value coloured by oceanographic structure (AC: anticyclone, C: cyclone, and TZ: transition zone). The direction and length of the arrows mark the direction and rate of steepest increase of the given significant environmental variable. Percentage variability along each axis is given in between parentheses.

The daytime CAP for echo-class 1 accounted for 63% of the total variability in the daytime acoustic data (Table 6). The first CAP axis (CAP1) accounted for 97% of the variance in daytime acoustic data of echo-class 1 and primarily separated the C from the AC and TZ (Fig 9a), with a strong positive correlation with fluorescence in the first 100 m (in the C), and a strong negative correlation with deeper fluorescence (100–200 m) (in the AC). The second axis (CAP2) accounted for only 3% of the variance in the dataset and the s_A_ values of echo-class 1 in the AC and TZ were driven by high EKE in the first 100 m (Table 6). The nighttime CAP1 axis of echo-class 1 showed strong negative correlations with fluorescence between 100–200 m and 15–100 m in the AC and TZ and explained 91% of the total variance. The CAP2 axis explained 9% of the total variance and showed a strong negative correlation with the mean EKE between 24–100 m in the TZ.

**Table 6:** Environmental predictor loadings for the daytime and nighttime CAP analyses of echo-classes 1 to 4. Predictors ≥ 0.6 on either of the first two CAP axes are shown in bold. Percentage variability is given in between parentheses.

Echo-class number	Environmental Predictors	CAP1	CAP2	CAP1	CAP2
		Day (63%)		Night (91%)	
Echo-class 1	EKE 24–100 m	−0.24	**0.97**	−0.05	**−1.00**
	Fluorescence 100–200 m	**−1.00**	−0.08	**−0.89**	−0.05
	Fluorescence 15–100 m	**0.89**	−0.35	**−0.68**	−0.34
Echo-class 2		Day and Night (24%)			
	EKE 24–100 m	0.04	**0.72**		
	Fluorescence 100–200 m	**0.67**	0.13		
	Salinity 15–100 m	**−0.79**	0.37		
	Salinity 200–250 m	0.43	−0.16		
Echo-class 3		Day (41%)		Night (55%)	
	EKE 200–248 m	−0.52	**0.82**	**0.67**	0.25
	Fluorescence 100–200 m	**−0.97**	0.16	0.32	**0.78**
	Salinity 100–200 m	**0.86**	0.05	**−0.84**	0.50
	Temperature 100–200 m	**−1.00**	0.06	**0.88**	−0.47
Echo-class 4		Day (64%)		Night (45%)	
	EKE 24–100 m	−0.20	**0.60**	−0.27	−0.35
	EKE 100–200 m	−0.59	0.31	−0.54	−0.50
	EKE 200–248 m	−0.56	0.47	**−0.66**	−0.18
	Fluorescence 15–100 m	**0.86**	−0.12	0.01	−0.05
	Fluorescence 100–200 m	**−0.96**	−0.09	−0.05	**−0.79**
	Fluorescence 200–250 m	**−0.87**	0.11	−0.05	−0.28
	Salinity 15–100 m	**0.96**	0.04	0.03	0.05
	Salinity 100–200 m	**0.97**	0.03	0.04	0.10
	Salinity 200–250 m	−0.33	−0.05	0.07	−0.03
	Temperature 15–100 m	**−0.92**	−0.01	0.17	−0.52
	Temperature 100–200 m	**−0.97**	−0.07	0.02	−0.28
	Temperature 200–250 m	**−0.96**	−0.05	0.12	−0.02

The CAP analysis explained 24% of the total variability in the daytime and nighttime s_A_ values of echo-class 2, with the first axis (CAP1) accounting for most of the variance (99%) and showing a strong negative correlation with salinity between 15–100 m and a positive correlation with fluorescence between 100–200 m in the AC and TZ (Fig 9b). The s_A_ values of echo-class 2 along CAP2 were largely driven by high EKE between 24 and 100 m in the AC and TZ (Table 6). The C was poorly represented in this CAP analysis due to a small number of data points.

Daytime s_A_ values of echo-class 3 showed a strong separation between the C driven positively by salinity between 100–200 m, and the AC and TZ showing strong negative correlations with temperature between 100–200 m along CAP1 which explained 98% of the variance (Fig 9c). Daytime s_A_ values of echo-class 3 in the AC and TZ were strongly positively driven by EKE between 200–248 m along CAP 2 which explained 1% of the variance (Table 6). The nighttime CAP of echo-class 3 explained 55% of the total variability compared to 41% of the variability in the daytime CAP. The C and AC showed a clear separation along CAP1 and CAP2 which explained 96% and 4% of the total variance, respectively. The s_A_ values of the echo-class 3 in the C were positively driven by fluorescence between 100–200 m along CAP2 and negatively by salinity between 100–200 m along CAP1. The s_A_ values of echo-class 3 in the AC were positively driven by EKE between 200–248 m and temperature between 100–200 m along CAP1.

The daytime CAP analysis accounted for 64% of the total variability in the s_A_ values of echo-class 4, with a clear separation along CAP1 between the C (driven positively by fluorescence and salinity between 15–200 m), and the AC and TZ (Fig 9d). Similarly to echo-classes 1 and 2, EKE between 24–100 m was strongly positively correlated with CAP2 in the AC and TZ. The daytime s_A_ values of echo-class 4 in the AC and TZ were further characterized by negative correlations with deep fluorescence (100–250 m) and shallow and deep temperature values (15–250 m) along CAP1 (Table 6). The nighttime s_A_ values of echo-class 4 in the C were separated from the AC, with the C being negatively driven by EKE 200–248 m along CAP1 and fluorescence 100–200 m along CAP2. The TZ was not represented in the nighttime CAP analysis of echo-classes 3 and 4 due to lack of matching temporal and spatial resolutions between the environmental and acoustic datasets.

#### 3.3.2 Biological significance of echo-classes

Since scattering models parametrized using values of speed of sound in the water at 25 m and 200 m depths, were classified into similar echo-classes, results are given only for the 25-m depth category in Table 7. The scattering models representing copepods (less than 3 mm), fluid-like organisms (3–5 mm), and euphausiids (3 and 4.6 mm) were classified into echo-class 1 (Table 7). Echo-class 2 was represented by organisms with small gas bubbles (0.16 mm). Echo-class 3 included gas-filled swimbladdered fish (25−185 mm), siphonophores with pneumatophore (0.5−1.5 mm), organisms with spherical and ellipsoid gas bubbles of varied sizes, and large euphausiids (10.5 mm). Echo-class 4 was represented by small siphonophores with pneumatophores (0.3 mm). A total of 47 models comprising small swimbladdered fish and siphonophores with pneumatophore (5 and 0.1 mm, respectively), copepods (0.05 to 0.77 mm), high pass fluid-sphere representing gastropods (0.5 to 2 mm), fluid-like organisms representing salps and shrimps, organisms with gas bubbles and euphausiids of a wide range of sizes were not classified into any of the 4 echo-classes (S3 File).

**Table 7:** Theoretical scattering models classified into the four echo-classes 1 to 4. The Escore value gives the relative position of each scattering model to the centroid of the echo-class. The smaller the Escore value, the closer the model is to the centroid of that echo-class.

Echo-class number	Model	ESR (mm)	Escore
Echo-class 1	Copepod (DWBA)	1.21	21
	Copepod (DWBA)	1.9	4
	Copepod (DWBA)	3	4
	Fluid, bent cylinder	3	9
	Fluid, bent cylinder	5	2
	Fluid, bent cylinder	3	9
	Fluid, bent cylinder	5	2
	Euphausiids (DWBA)	3	13
	Euphausiids (DWBA)	4.6	3
Echo-class 2	Gas bubble—spherical	0.16	8
Echo-class 3	Hybrid fish	25	2
	Hybrid fish	45	4
	Hybrid fish	65	4
	Hybrid fish	85	4
	Hybrid fish	105	4
	Hybrid fish	125	3
	Hybrid fish	145	2
	Hybrid fish	165	1
	Hybrid fish	185	1
	Hybrid siphonophore	0.5	7
	Hybrid siphonophore	0.7	0
	Hybrid siphonophore	0.9	2
	Hybrid siphonophore	1.1	3
	Hybrid siphonophore	1.3	3
	Hybrid siphonophore	1.5	3
	Gas bubble—ellipsoid	0.53	16
	Gas bubble—ellipsoid	0.95	18
	Gas bubble—ellipsoid	1.71	11
	Gas bubble—ellipsoid	3.08	18
	Gas bubble—ellipsoid	10	17
	Gas bubble—spherical	0.95	12
	Gas bubble—spherical	1.71	15
	Gas bubble—spherical	5.55	23
	Gas bubble—spherical	10	5
	Large euphausiids (DWBA)	10.5	19
Echo-class 4	Hybrid siphonophore	0.3	1

## 4. Discussion

Previous studies investigated the influence of eddies and fronts on overall mesopelagic communities at a few working frequencies [5, 10, 25, 26, 39, 46]. This study used a four-frequency approach to identify acoustic groups potentially representing several types of mesopelagic organisms, and investigated the influence of an AC, C and the TZ between the two eddies on their distributions. We describe the application of the Escore algorithm on multifrequency echosounder data for the first time, and the open-source code is made available to facilitate future implementations of the method.

### 4.1 The Escore algorithm relative to other multifrequency backscatter classification approaches

The echosounder frequency and size, shape, orientation and material properties of marine organisms strongly influence acoustic echo levels [47]. Previous multifrequency methods developed over years of collecting and analysing multifrequency acoustic data classified the following groups of scatterers: gas-bearing organisms (fishes with swimbladder, and siphonophores with pneumatophores), fluid-like zooplankton (amphipods, euphausiids, decapods, gelatinous, jellyfish, squids, and fishes without swimbladders), and elastic shelled organisms such as pteropods, across many environmental and biological conditions [7, 9, 12]. Broad categories of scatterers have distinct frequency response curves in the 10–200 kHz frequency band [9]. The relative frequency response has been widely used as a basic indicator for attributing acoustic backscatter energy to the broad categories of scatterers [8, 10] over a wide range of animal sizes (or acoustic frequencies) [12, 34, 37, 48].

The Escore algorithm has similarities to previous multifrequency methods for backscatter classification. RGB echograms are useful triple-frequency visualization tools to determine the vertical patterns of organisms responding at several frequencies [11]. The concept of combining two objective functions, namely a supervised ROI selection from inspection of echograms, and an unsupervised clustering approach is similar to previous work on semi-supervised classification of multifrequency data (e.g., [49–51]). [13] also divided echograms into segments, calculated several metrics to describe the distribution of acoustic density in each segment and used ordination and clustering to assign the echogram segments to a reduced number of categories or clusters. [14] used the SHAPES (shoal analysis and patch estimation system) algorithm [52] to select discrete schools and calculated frequency sums and a classification tree. [53] also used a ROI detection step and ROI classification to distinguish Atlantic herring schools in multi-frequency echograms. In the absence of discrete schools within our oceanographic environment, we developed the ROI selection step to isolate acoustic structures with homogeneous frequency responses.

The Escore approach uses clustering methods on pairwise frequency differences (characterized by normalizing the backscatter to the 38 kHz reference frequency by convention) [12] to classify scatterers into distinct acoustic groups. Similar to [8], the distribution of cell-averaged ΔS_v_ is assumed to approximate a normal distribution and the deviations of an observed frequency difference from a frequency pair observed in any sample, is summarized by the normal deviate or Z-score. Following the suggestion of [8], as a measure of refinement of the Z-score approach to maximize the probability of discrimination between scatterers, four frequencies were used in Step 1 of the Escore method as a pre-processing step to isolate biological structures of interest before conducting a K-means clustering at frequency pairs at which these structures differ. K-means are the most commonly used clustering techniques which partition data into a predetermined number of clusters with the aim to minimize the dissimilarity within groups [12]. While choosing the number of clusters *a priori* and the inability to deal with outliers are often seen as drawbacks of K-means, we leveraged these aspects to our advantage by selecting only 1 cluster within each ROI to represent a single coherent biological structure.

While the Escore algorithm does not solve “the grand challenge” of identification of organisms to the species level using multifrequency acoustic data [54], it may provide substantial insight into the vertical distribution of scatterers in the water column, especially when combined with other multifrequency acoustic visualization tools (such as RGB echograms), statistical techniques and theoretical scattering models fine-tuned with trawl observations if available. Furthermore, this approach can be applied using different frequencies, for varied scientific objectives and/or in various oceanographic environments.

### 4.2 Influence of mesoscale eddies and fronts on the distribution of mesopelagic organisms

It has been well documented in various oceans that water mass properties in mesoscale eddies and fronts influence the distribution and composition of mesopelagic organisms, including micronekton: Mid-Atlantic Ridge [46], Canary Archipelago [55, 56], South West Indian Ocean [5, 24–26], northern Gulf of Mexico [43], north Atlantic [57–59], eastern Pacific [39], western North Pacific subtropical gyre [60], south Pacific [61], and Southern Ocean [62]. Mesoscale eddies create thermal niches that support growth of different species, enhance productivity which can possibly lead to greater feeding opportunities [58, 59, 63], and act as “oases” [46] or “buses” by trapping and transporting micronekton inside their core [57], compared to the surrounding waters.

While studies showed greater micronekton acoustic densities in cyclones compared to anticyclones [24], others observed greater densities in anticyclones [18, 46, 58]. The warm cores of anticyclonic eddies are believed to increase the metabolic rates of micronekton [58], likely increasing biomass [64]. Studies showing greater micronekton acoustic densities in cyclones also showed these systems to be more productive than anticyclones [24]. Consistent with previous observations in the Mozambique Channel, the C encountered in our study was more productive than the AC and showed greater daytime total NASC at the 18, 38 and 70 kHz, and nighttime NASC at the 18, 70, 120 and 200 kHz frequencies. The AC showed greater daytime total NASC than the C at higher frequencies (120 and 200 kHz) and greater nighttime NASC at the 38 kHz. These observations demonstrate that different organisms, or similar organisms but of different sizes are influenced differently by cyclones and anticyclones depending on the time of day. Some groups of organisms seem to be concentrated mostly within the C and others in the AC of the Mozambique Channel during daytime or nighttime.

The DVM pattern of mesopelagic organisms was shown by the higher NASC observed in the surface layer during nighttime in the AC, C and TZ at all frequencies compared to daytime, similar to previous studies [2, 5, 65]. The DVM signal was different at different frequencies in the AC, C and TZ despite these areas being spatially close to each other. The cores of eddies showed greater total NASC and proportion of migrating organisms than the TZ, at most frequencies. An inverse migration pattern (with greater NASC in the surface layer during the day compared to the deep layer) in the C which was previously observed in the South West Indian Ocean and was related to enhanced feeding opportunities for micronekton and/or reduction in competition with other conspecifics [24], was not apparent in this study. Eddies of the South West Indian Ocean therefore show varied influences on the DVM of mesopelagic organisms, likely related to eddy properties such as the formation, intensity, age, duration, and eddy-induced Ekman pumping [66]. Eddy influences on DVM may also be related to the biological properties of organisms such as community structure, swimming patterns and speeds, and responsiveness of organisms to physical oceanographic variables.

### 4.3 Influence of eddies and front on the distribution of acoustic groups

Preliminary observations indicate that small fluid-like organisms such as copepods and euphausiids are potential candidates for the frequency response curve of echo-class 1 which shows an increasing relative frequency response between 18 and 38 kHz [38, 67, 68]. The echograms of echo-class 1 in the C showed a strong association with the fluorescence maximum depth between 50 and 100 m. The potential zooplankton comprising echo-class 1 may be following their phytoplanktonic prey.

The only potential candidate for echo-class 2 are organisms with small gas bubbles showing a strong resonance at 38 kHz, possibly corresponding to small siphonophores and small gas-bearing mesopelagic fishes of juvenile stages [69]. Furthermore, echo-class 2 showed no apparent vertical stratification between the AC, C, and TZ, but with a marked preference for the AC during daytime and AC and TZ during nighttime (as shown by total NASC values). The vertical distributions of the small siphonophores and small gas-bearing fishes of juvenile stages comprising echo-class 2 may be sensitive to the vertical salinity barriers in the mesoscale structures as shown by the echograms, vertical section of the MVP data (Fig 7), and the GAMM plot (Fig 8).Organisms with gas bubbles, siphonophores with pneumatophores and gas-filled swimbladdered mesopelagic fish (larger than those showing a resonance at 38 kHz) may present high backscatter values at 18 kHz [8, 10, 70] potentially consistent with echo-class 3. Compared to the other echo-classes, echo-class 3 showed a higher total NASC within the C relative to the AC and TZ both during daytime and nighttime. It was mainly influenced by salinity, temperature and fluorescence within the intermediate 100–200 depth range and mean EKE at deeper depths (200–248 m) compared to the other echo-classes. As shown by the echograms, some of the organisms comprising echo-class 3 migrated vertically downward at sunrise while some remained at the surface during daytime. A great proportion of mesopelagic fishes are known to be diel vertical migrants [70] and may likely be some of the potential candidates of echo-class 3.

Siphonophores of smaller sizes than those of echo-class 3, are potential candidates belonging to echo-class 4 that appear to be influenced differently by the vertical gradients of temperature and salinity, compared to the other echo-classes. Depending on their size and depth, gas-bearing organisms such as siphonophores and pyrosomes may present a maximum echo at the 38 kHz [70–72], as observed in this study.

Eddies have been associated with distinct zooplankton communities and assemblages [73] which are attractive to different micronekton communities, hence the varied NASC between physical oceanographic structures and at different frequencies. These observations support an emerging understanding of dynamic behavioural responses of predators with respect to their prey, which leads to the restructuring of the zonation of mesopelagic organisms [74, 75]. Studies have also shown that mesopelagic organisms have different affinities for water masses with distinct temperature, salinity, chlorophyll and sea surface height gradients [39, 43, 62], consistent with findings of this study whereby the four echo-classes, representing gas-bearing and fluid-like organisms, were structured differently in the AC, C, and TZ according to the depths of the thermocline, halocline and fluorescence maximum. A strong correlation between the acoustic densities of echo-classes (especially echo-class 4 which potentially represent small siphonophores) and environmental variables at intermediate (100–200 m) and deeper depths (greater than 250 m) is consistent with the downwelling processes that characterise anticyclones. We cannot exclude the potential influence of other environmental drivers not examined here, such as dissolved oxygen, light intensity, and nutrients on the distribution of mesopelagic organisms [39, 62, 76].

Studies have suggested that micronekton are generally lethargic when not migrating or escaping from a threat [77] and only initiate movement in response to a stimulus such as gradients in temperature, pressure or light [63]. From the inner core of a mesoscale eddy, micronekton inhabiting deeper layers during daytime must be able to perceive and respond to gradients on scales that are several orders of magnitudes larger than their body size since the closest horizontal gradients in these structures are tens or hundreds of kilometres above [63], with higher interactions between eddies and micronekton occurring during nighttime after organisms have migrated. Eddy persistence and duration over several days and eddy recurrence in the Mozambique Channel [78] can allow for sufficient time for micronekton to respond to physical oceanographic changes in their environment. The echo-classes in the TZ were highly correlated to mean EKE within the first 250 m of the water column. TZ usually show strong current speeds extending to 400 m [24]. These zones aggregate distinct communities of smaller and weaker swimmers not being able to traverse these strong barriers [73]. This study shows that in the TZ, echo-classes 3 and 4 were vertically structured according to temperature, salinity and fluorescence gradients. In contrast to [59], we showed that the TZ may possibly attract a small proportion of stronger swimmers such as organisms with large gas bubbles peaking at 18 kHz (echo-class 3). Drifting particles such as plankton are likely to concentrate in the frontal structures and be carried along in the current flow within these zones (Fig 7c) [16], thereby possibly attracting these larger-sized diel vertical migrants.

## 5. Conclusion

Biological resources in the ocean are non-uniformly distributed across various spatial and temporal scales [79] and are modulated by a variety of processes including mesoscale features such as eddies and fronts. Ocean warming and subsequent decline in chlorophyll is predicted to impact the dynamics of mesoscale eddies [80], decrease the biomass and deepen micronekton in the tropics [81]. Current knowledge gaps regarding how specific groups of mesopelagic organisms are influenced by mesoscale eddies limit our understanding of how changes in eddy dynamics may impact populations [59], and is of particular relevance in the context of a warming ocean and interests in the commercial exploitation of these organisms. This study used a multi-frequency acoustic backscatter classification approach to investigate the influence of mesoscale eddies on mesopelagic organisms. We demonstrated the potential of the Escore algorithm in a real-world test case. Mesoscale eddies are common features in the Mozambique Channel and regularly interact with mesopelagic organisms along their paths. The mesopelagic composition of the sampled mesoscale eddies in the Mozambique Channel is heterogeneous, with no particular type of scatterer dominating at all frequencies. The varying migration patterns of mesopelagic organisms also changed the relative day/night abundances of different groups at any given depth, potentially affecting the balance of dominant scatterers. This study showed a link between the physical processes associated with eddies and fronts and the biological response of different groups of mesopelagic organisms. We suggest using the Escore algorithm as a tool with validation from trawl catches, eDNA presence/absence data, and/or theoretical scattering models in the absence of trawl and eDNA data, to discriminate and classify multi-frequency acoustic backscatter into several groups representing various mesopelagic organisms, even in physical oceanographic environments which are heterogeneous.

## Supporting information

S1 File Data classification according to oceanographic structures. (DOCX)

S2 File Implementation of the Escore algorithm. (DOCX)

S3 File Description of theoretical scattering models. (DOCX)

S4 File Acoustic modelling—GAMM. (DOCX)

S5 File Testing for collinearity between environmental covariates. (DOCX)

## References

[1] Proud R, Cox M J, Brierley A S. Biogeography of the Global Ocean’s Mesopelagic Zone. Current Biology. 2017;27:113–119. doi: 10.1016/j.cub.2016.11.003 28017608

[2] Cotté C, Ariza A, Berne A, Habasque J, Lebourges-Dhaussy A, Roudaut G, et al. Macrozooplankton and micronekton diversity and associated carbon vertical patterns and fluxes under distinct productive conditions around the Kerguelen Islands. Journal of Marine Systems. 2022;226:103650.

[3] De Forest L, and Drazen J. The influence of a Hawaiian seamount on mesopelagic micronekton. Deep Sea Research Part I: Oceanographic Research Papers, 2009;56:232–250.

[4] Potier M, Bach P, Ménard F, Marsac F. Influence of mesoscale features on micronekton and large pelagic fish communities in the Mozambique Channel. Deep Sea Research Part II: Topical Studies in Oceanography. 2014;100:184–199.

[5] Annasawmy P, Ternon JF, Marsac F, Cherel Y, Béhagle N, Roudaut G, et al. Micronekton diel migration, community composition and trophic position within two biogeochemical provinces of the South West Indian Ocean: Insight from acoustics and stable isotopes. Deep Sea Research Part I: Oceanographic Research Papers. 2018;138:85–97.

[6] Le Moigne FAC. Pathways of Organic Carbon Downward Transport by the Oceanic Biological Carbon Pump. Frontiers in Marine Science. 2019;6:634.

[7] Fernandes PG, Korneliussen RJ, Lebourges-Dhaussy A, Masse J, Iglesias M, Diner N, et al. The SIMFAMI Project: Species Identification Methods from Acoustic Multifrequency Information. Final Report to the EC, Q5RS-2001–02054. 2006.

[8] De Robertis A, McKelvey DR, Ressler PH. Development and application of an empirical multifrequency method for backscatter classification. Canadian Journal of Fisheries and Aquatic Sciences. 2010;67:1459–1474.

[9] Trenkel VM, Berger L. A fisheries acoustic multi-frequency indicator to inform on large scale spatial patterns of aquatic pelagic ecosystems. Ecological Indicators. 2013;30:72–79.

[10] Béhagle N, Cotté C, Lebourges-Dhaussy A, Roudaut G, Duhamel G, Brehmer P, et al. Acoustic distribution of discriminated micronektonic organisms from a bi-frequency processing: The case study of eastern Kerguelen oceanic waters. Progress in Oceanography. 2017;156:276–289.

[11] Annasawmy P, Ternon JF, Cotel P, Cherel Y, Romanov EV, Roudaut G, et al. Micronekton distributions and assemblages at two shallow seamounts of the south-western Indian Ocean: Insights from acoustics and mesopelagic trawl data. Progress in Oceanography. 2019;178:102161.

[12] Korneliussen RJ, et al. Acoustic target classification. ICES Cooperative Research Report. 314, Copenhagen, Denmark. 2018.

[13] Burgos JM, Horne JK. Characterization and classification of acoustically detected fish spatial distributions. ICES Journal of Marine Science. 2008. 65:1235–1247.

[14] Fernandes PG. Classification trees for species identification of fish-school echotraces. ICES Journal of Marine Science. 2009;66:1073–1080. doi: 10.1093/icesjms/fsp060

[15] Tew-Kai E, Marsac F. Patterns of variability of sea surface chlorophyll in the Mozambique Channel: A quantitative approach. Journal of Marine Systems. 2009;77:77–88.

[16] Hancke L, Roberts MJ, Ternon JF. Surface drifter trajectories highlight flow pathways in the Mozambique Channel. Deep Sea Research Part II: Topical Studies in Oceanography. 2014;100:27–37.

[17] Vinayachandran PNM, Masumoto Y, Roberts M, Huggett J, Halo I, Chatterjee A, et al. Reviews and syntheses: Physical and biogeochemical processes associated with upwelling in the Indian Ocean. Biogeosciences. 2021;18(22):5967–6029. doi: 10.5194/bg-18-5967-2021

[18] Arostegui MC, Gaube P, Woodworth-Jefcoats PA, Kobayashi DR, Braun CD. Anticyclonic eddies aggregate pelagic predators in a subtropical gyre. Nature. 2022;609(7927): 535–540. https://www.nature.com/articles/s41586-022-05162-6. 36071164 10.1038/s41586-022-05162-6

[19] Balwada D, Xie JH, Marino R, Feraco F. Direct observational evidence of an oceanic dual kinetic energy cascade and its seasonality. Science Advances. 2022;8(41):eabq2566. http://arxiv.org/abs/2202.08637. 36223461 10.1126/sciadv.abq2566 PMC9555769

[20] Quartly GD, Srokosz MA. Eddies in the southern Mozambique Channel. Deep Sea Research Part II: Topical Studies in Oceanography. 2004;51:69–83.

[21] de Ruijter WPM, van Aken HM, Beier EJ, Lutjeharms JRE, Matano RP, Schouten MW. Eddies and dipoles around South Madagascar: formation, pathways and large-scale impact. Deep Sea Research Part I: Oceanographic Research Papers. 2004;51:383–400.

[22] Roberts MJ, Ternon JF, Morris T. Interaction of dipole eddies with the western continental slope of the Mozambique Channel. Deep Sea Research Part II: Topical Studies in Oceanography. 2014;100:54–67.

[23] Huggett JA. Mesoscale distribution and community composition of zooplankton in the Mozambique Channel. Deep Sea Research Part II: Topical Studies in Oceanography. 2014;100: 119–135.

[24] Annasawmy P, Ternon JF, Lebourges-Dhaussy A, Roudaut G, Cotel P, Herbette S, et al. Micronekton distribution as influenced by mesoscale eddies, Madagascar shelf and shallow seamounts in the south-western Indian Ocean: an acoustic approach. Deep Sea Research Part II: Topical Studies in Oceanography. 2020;176:104812.

[25] Sabarros P, Ménard F, Lévénez J, Tew-Kai E, Ternon J. Mesoscale eddies influence distribution and aggregation patterns of micronekton in the Mozambique Channel. Marine Ecology Progress Series. 2009;395:101–107.

[26] Béhagle N, du Buisson L, Josse E, Lebourges-Dhaussy A, Roudaut G, Ménard F. Mesoscale features and micronekton in the Mozambique Channel: An acoustic approach. Deep Sea Research Part II: Topical Studies in Oceanography. 2014;100:164–173.

[27] Le Bot P, Kermabon C, Lherminier P, Gaillard F. CASCADE V6.1: Logiciel de validation et de visualisation des mesures ADCP de coque. Rapport technique OPS/LPO 11–01. Ifremer, Centre de Brest, France. 2011.

[28] Demer DA, Berger L, Bernasconi M, Bethke E, Boswell K, Chu D, et al. Calibration of acoustic instruments. ICES Cooper Res Rep 326:133. 2015.

[29] Perrot Y, Brehmer P, Habasque J, Roudaut G, Behagle N, Sarré A, et al. Matecho: An Open-Source Tool for Processing Fisheries Acoustics Data. Acoustics Australia. 2018;46:241–248.

[30] De Robertis A, Higginbottom I. A post-processing technique for estimation of signal-to-noise ratio and removal of echosounder background noise. ICES J Mar Sci. 2007;64:1282–1291.

[31] Ryan TE, Downie RA, Kloser RJ, Keith G. Reducing bias due to noise and attenuation in open-ocean echo integration data. ICES Journal of Marine Science. 2015;72:2482–2493.

[32] Korneliussen RJ, Ona E. An operational system for processing and visualizing multi-frequency acoustic data. ICES Journal of Marine Science. 2002;159:293–313.

[33] Charrad M, Ghazzali N, Boiteau V, Niknafs A. NbClust: An R package for determining the relevant number of clusters in a data set. J Stat Softw. 2014;61:1–36.

[34] Stanton TK, Chu D. Review and recommendations for the modelling of acoustic scattering by fluid-like elongated zooplankton: euphausiids and copepods. ICES Journal of Marine Science. 2000;57(4):793–807. jmsc.1999.0517

[35] Lavery AC, Wiebe PH, Stanton TK. Determining dominant scatterers of sound in mixed zooplankton populations. The Journal of the Acoustical Society of America. 2007;122:3304. doi: 10.1121/1.2793613 18247742

[36] Stanton T, Wiebe PH, Chu D, Benfield M., Scanlon L, Martin L, et al. On acoustic estimates of zooplankton biomass. ICES (Int. Counc. Explor. Sea) J. Mar. Sci. 1994;51:505–512. doi: 10.1006/jmsc.1994.1051

[37] Barbin L, Lebourges-Dhaussy A, Allain V, Receveur A, Lehodey P, Habasque J, et al. Comparative analysis of day and night micronekton abundance estimates in west Pacific between acoustic and trawl surveys. Deep Sea Research Part I: Oceanographic Research Papers. 2024;204:104221. doi: 10.1016/j.dsr.2023.104221

[38] Stanton TK, Chu D, Wiebe PH, Martin LV, Eastwood RL. Sound scattering by several zooplankton groups. II. Scattering models. The Journal of the Acoustical Society of America. 1998;103: 236–53. doi: 10.1121/1.421110 9440326

[39] Perelman JN, Ladroit Y, Escobar-Flores P, Firing E, Drazen JC. Eddies and fronts influence pelagic communities across the eastern Pacific Ocean. Progress in Oceanography. 2023;211:102967.

[40] Oksanen J, Blanchet FG, Friendly M, Kindt R, Legendre P, McGlinn D, et al. vegan: community ecology package (version 2.5.6). 2019. https://CRAN.R-project.org/package=vegan

[41] Wood SN. Fast stable restricted maximum likelihood and marginal likelihood estimation of semiparametric generalized linear models. J R Stat Soc. 2011;73:3–36.

[42] Receveur A. Ecologie spatiale du micronecton: distribution, diversité et importance dans la structuration de l’écosystème pélagique du Pacifique sud-ouest. Doctoral Thesis. Aix-Marseille University. 2019.

[43] Boswell KM, D’Elia M, Johnston MW, Mohan JA, Warren JD, Wells RJD, et al. Oceanographic Structure and Light Levels Drive Patterns of Sound Scattering Layers in a Low-Latitude Oceanic System. Frontiers in Marine Science. 2020;7:51. doi: 10.3389/fmars.2020.00051

[44] Dornan T, Fielding S, Saunders RA, Genner MJ. Large mesopelagic fish biomass in the Southern Ocean resolved by acoustic properties. Proceedings of the Royal Society B: Biological Sciences. 2022;289:20211781. doi: 10.1098/rspb.2021.1781 35078354 PMC8790350

[45] Anderson MJ, Willis TJ. Canonical analysis of principal coordinates: a useful method of constrained ordination for ecology. J. Appl. Ecol. 2003;84:511–525. doi: 10.1111/j.1526-100X.2005.00051.x

[46] Godø OR, Samuelsen A, Macaulay GJ, Patel R, Hjøllo SS, Horne J, et al. Mesoscale Eddies Are Oases for Higher Trophic Marine Life. PLoS ONE. 2012;7:e30161. doi: 10.1371/journal.pone.0030161 22272294 PMC3260222

[47] Stanton T, Chu D, Wiebe PH. Acoustic scattering characteristics of several zooplankton groups. ICES Journal of Marine Science. 1996;53:289–295.

[48] Jech JM, Lawson GL, Lowe MR. Comparing acoustic classification methods to estimate krill biomass in the Georges Bank region from 1999 to 2012: Georges Bank krill biomass. Limnology and Oceanography: Methods. 2018;16:680–695.

[49] Korneliussen RJ, Heggelund Y, Macaulay GJ, Patel D, Johnsen E, Eliassen IK. Acoustic identification of marine species using a feature library. Methods in Oceanography. 2016;17:187–205.

[50] Woillez M, Ressler PH, Wilson CD, Horne JK. Multifrequency species classification of acoustic-trawl survey data using semi-supervised learning with class discovery. JASA Express Letters. 2012;131(2):EL184. doi: 10.1121/1.3678685 22352620

[51] Choi C, Kampffmeyer M, Handegard NO, Salberg AB, Jenssen R. Deep Semisupervised Semantic Segmentation in Multifrequency Echosounder Data. IEEE Journal of Oceanic Engineering. 2023;1–17.

[52] Coetzee J. Use of a shoal analysis and patch estimation system (SHAPES) to characterise sardine schools. Aquat Living Resour. 2000;13:1–10. doi: 10.1016/S0990-7440(00)00139-X

[53] Zhang Y, Wall CC, Jech JM, Lv Q. Developing a hybrid model with multiview learning for acoustic classification of Atlantic herring schools. Limnology and Oceanography: Methods. 2024;1–18. doi: 10.1002/lom3.10611

[54] MacLennan DN, Holliday DV. Fisheries and plankton acoustics: past, present, and future. ICES Journal of Marine Science. 1996;53:513–516.

[55] Ariza A, Garijo JC, Landeira JM, Bordes F, Hernández-León S. Migrant biomass and respiratory carbon flux by zooplankton and micronekton in the subtropical northeast Atlantic Ocean (Canary Islands). Progress in Oceanography. 2015;134: 330–342.

[56] Peña M, Vélez-Belchí P. Shaping of deep scattering layers by the seascape dynamics in the Canary Islands. Progress in Oceanography. 2023;210:102953. doi: 10.1016/j.pocean.2022.102953

[57] Fennell S, Rose G. Oceanographic influences on Deep Scattering Layers across the North Atlantic. Deep Sea Research Part I: Oceanographic Research Papers. 2015;105:132–141.

[58] Della Penna A, Gaube P. Mesoscale Eddies Structure Mesopelagic Communities. Frontiers in Marine Science. 2020;7:454.

[59] Devine B, Fennell S, Themelis D, Fisher JAD. Influence of anticyclonic, warm-core eddies on mesopelagic fish assemblages in the Northwest Atlantic Ocean. Deep Sea Research Part I: Oceanographic Research Papers. 2021;173:103555.

[60] Wang Y, Zhang J, Yu J, Wu Q, Sun D. Anticyclonic mesoscale eddy induced mesopelagic biomass hotspot in the oligotrophic ocean. Journal of Marine Systems. 2023;237:103831. doi: 10.1016/j.jmarsys.2022.103831

[61] Domokos R. Environmental effects on forage and longline fishery performance for albacore (*Thunnus alalunga*) in the American Samoa Exclusive Economic Zone. Fisheries Oceanography. 2009;18:6. doi: 10.1111/j.1365-2419.2009.00521.x

[62] Woods BL, Van de Putte AP, Hindell MA, Raymond B, Saunders RA, Walters A, et al. Species distribution models describe spatial variability in mesopelagic fish abundance in the Southern Ocean. Frontiers in Marine Science. 2023;9:981434.

[63] Della Penna A, Llort J, Moreau S, Patel RS, Kloser RJ, Gaube P, et al. The impact of a Southern Ocean cyclonic eddy on mesopelagic micronekton. Journal of Geophysical Research: Oceans. 2022;127:e2022JC018893. doi: 10.1029/2022JC018893

[64] Keates TR, Hazen EL, Holser RR, Fiechter J, Bograd SJ, Robinson PW, et al. Foraging behavior of a mesopelagic predator, the northern elephant seal, in northeastern Pacific eddies. Deep Sea Research Part I: Oceanographic Research Papers. 2022;189:103866.

[65] Wiebe PH, Lavery AC, Lawson GL. Biogeographic variations in diel vertical migration determined from acoustic backscattering in the northwest Atlantic Ocean. Deep Sea Research Part I: Oceanographic Research Papers.2022;103887.

[66] Benitez-Nelson CR, McGillicuddy DJ. Mesoscale physical—biological—biogeochemical linkages in the open ocean: An introduction to the results of the E-Flux and EDDIES programs. Deep Sea Research Part II: Topical Studies in Oceanography. 2008;55:1133–1138.

[67] Lavery AC, Stanton TK, McGehee DE, Chu D. Three-dimensional modeling of acoustic backscattering from fluid-like zooplankton. The Journal of the Acoustical Society of America. 2002;111:1197–1210. doi: 10.1121/1.1433813 11931297

[68] Korneliussen R, Ona E. Synthetic echograms generated from the relative frequency response. ICES Journal of Marine Science. 2003;60:636–640.

[69] Blanluet A, Doray M, Berger L, Romagnan JB, Le Bouffant N, Lehuta S, et al. Characterization of sound scattering layers in the Bay of Biscay using broadband acoustics, nets and video. PLoS One. 2019;14:e0223618. doi: 10.1371/journal.pone.0223618 31634351 PMC6802824

[70] Ariza A, Landeira JM, Escánez A, Wienerroither R, Aguilar de Soto N, Røstad A, et al. Vertical distribution, composition and migratory patterns of acoustic scattering layers in the Canary Islands. Journal of Marine Systems. 2016;157:82–91.

[71] Cotter E, Bassett C, Lavery A. Classification of broadband target spectra in the mesopelagic using physics-informed machine learning. The Journal of the Acoustical Society of America. 2021;149:3889–3901. doi: 10.1121/10.0005114 34241451

[72] Ariza A, Lebourges‐Dhaussy A, Nerini D, Pauthenet E, Roudaut G, Assunção R, et al. Acoustic seascape partitioning through functional data analysis. Journal of Biogeography. 2022. doi: 10.1111/jbi.14534

[73] Gibbons MJ, Parker Y, Cedras RB, Thibault D. Mesoscale structure of neuston assemblages across the southern Indian Ocean subtropical gyre. Deep Sea Research Part II: Topical Studies in Oceanography. 2023;208:105249.

[74] Urmy SS, Benoit-Bird KJ. Fear dynamically structures the ocean’s pelagic zone. Current Biology. 2021;31:5086–5092.e3. doi: 10.1016/j.cub.2021.09.003 34562382

[75] Grassian B, Roman C, Omand M, Wishner K, Seibel B. Multi-sensor observation of a rapidly dispersing micronekton thin layer. Deep Sea Research Part I: Oceanographic Research Papers. 2022;103924.

[76] Song Y, Yang J, Wang C, Sun D. Spatial patterns and environmental associations of deep scattering layers in the northwestern subtropical Pacific Ocean Acta Oceanol. Sin, 2022;41:1–14.

[77] Kaartvedt S, Staby A, Aksnes D. Efficient trawl avoidance by mesopelagic fishes causes large underestimation of their biomass. Marine Ecology Progress Series. 2012;456: 1–6.

[78] Sudre F, Dewitte B, Mazoyer C, Garçon V, Sudre J, Penven P, et al. Spatial and seasonal variability of horizontal temperature fronts in the Mozambique Channel for both epipelagic and mesopelagic realms. Frontiers in Marine Science. 2023;9:1045136. doi: 10.3389/fmars.2022.1045136

[79] Haury LR, McGowan JA, Wiebe PH. Patterns and processes in the time-space scales of plankton distributions. In: Steele JH (ed) Spatial pattern in plankton communities. Springer, Boston, pp 277–327.1978.

[80] Beech N, Rackow T, Semmler T, Danilov S, Wang Q, Jung T. Long-term evolution of ocean eddy activity in a warming world. Nature Climate Change. 2022;12:910–917.

[81] Ariza A, Lengaigne M, Menkes C, Lebourges-Dhaussy A, Receveur A, Gorgues T, et al. Global decline of pelagic fauna in a warmer ocean. Nature Climate Change. 2022;12:928–934. https://www.nature.com/articles/s41558-022-01479-2.

