# Supplementary material for: Impact of an eddy dipole of the Mozambique channel on mesopelagic organisms, highlighted by multifrequency backscatter classification
Source: PLoS One. 2024 Sep 11;19(9):e0309840. doi: 10.1371/journal.pone.0309840 (PMC12139656; doi:10.1371/journal.pone.0309840)

**S1 Data classification according to oceanographic structures**

The sea surface temperature (SSTP) in °C and sea surface practical salinity (SSPS) recorded along the ship track by the thermosalinograph were used to classify the acoustic and environmental data points according to their location with respect to the anticyclone (AC), cyclone (C), and transition zone (TZ). The sea surface temperature and sea surface salinity clearly identify a density front at the interface of the TZ and C as shown in the figure below.


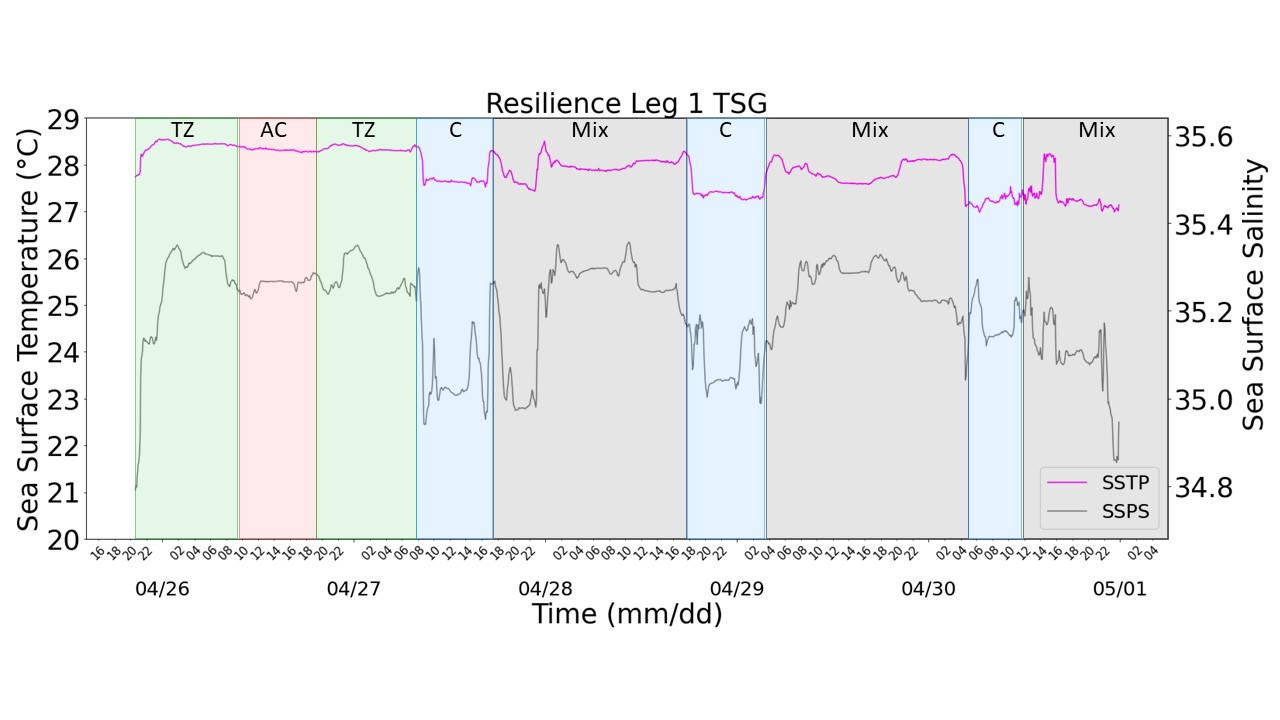

Supplement: S1 File — (DOCX) [file pone.0309840.s001.docx]
