# Supplementary material for: Impact of an eddy dipole of the Mozambique channel on mesopelagic organisms, highlighted by multifrequency backscatter classification
Source: PLoS One. 2024 Sep 11;19(9):e0309840. doi: 10.1371/journal.pone.0309840 (PMC12139656; doi:10.1371/journal.pone.0309840)
Supplement: S2 File — (DOCX) [file pone.0309840.s002.docx]

**S2 Implementation of the Escore algorithm**

Steps 1 to 5 were implemented to train the algorithm and step 6 to apply the algorithm to the whole acoustic dataset:

**Step (1)** A multi-frequency acoustic visualization approach based on RGB (red, green, blue) color coding was used to generate an RGB echogram in Matlab v. 9.11.0. 1837725 (c.f. Annasawmy *et al.*, 2019). The S_v_ values of the 18 kHz frequency was color-coded in red; 38 kHz in green; and 70 kHz in blue for the 18/38/70 kHz RGB echogram. For the 38/70/120 kHz echogram (S2 Fig. 1), the S_v_ values of the 38 kHz frequency was color-coded in red; 70 kHz in green; and 120 kHz in blue. A total of 77 rectangular regions of interests (ROI) were manually selected from the RGB echogram at 18/38/70 kHz, and 138 ROI from the RGB echogram at 38/70/120 kHz from the surface to 250 m. These ROI include at least one visible structure with a homogeneous frequency response in a layer with coherent acoustic and spatial characteristics (S2 Fig. 2a). The 200 kHz frequency was not used since it has the lowest range (only 125 m) of all the available frequencies.


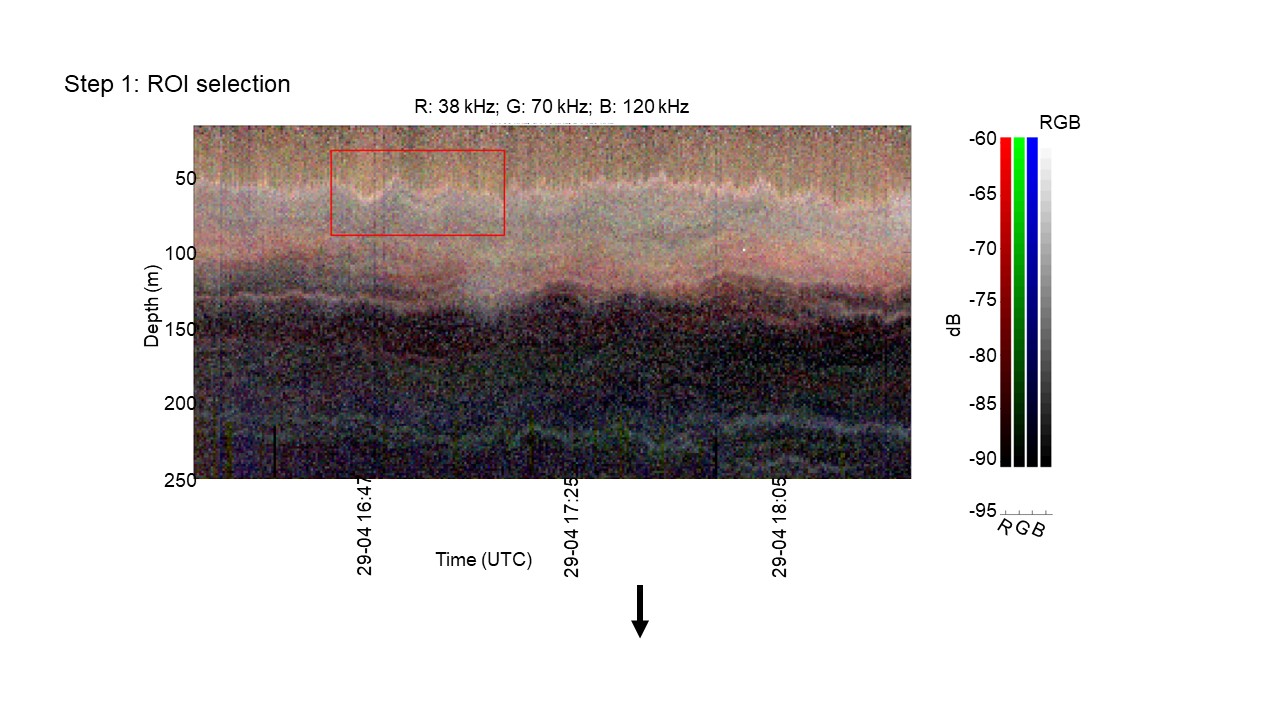


*Figure 1. RGB echogram at 38, 70 and 120 kHz showing the selection of one ROI (bounded by the red outlined rectangle). The color bar shows S_v_ (dB) at each corresponding frequency (Red (R): 38 kHz; Green (G): 70 kHz; Blue (B): 120 kHz).*

This step is analogous to previously published semi-supervised methods which used the SHAPES (shoal analysis and patch estimation system) algorithm on segmented sections of echograms to isolate discrete schools (Burgos and Horne 2008; Fernandes, 2009). Since layers of organisms were observed in our echograms (S2 Fig. 1) instead of discrete schools, we developed this semi-supervised approach by selecting ROI with coherent acoustic and spatial characteristics which will be further discriminated in Step 2.

**Step (2)** The pixels within each ROI were subjected to a K-means clustering based on the frequency responses at 18, 70, and 120 kHz relative to the 38 kHz (ΔS_v,18−38_, ΔS_v,70−38_, and ΔS_v,120−38_) in dB (S2 Fig. 2) by setting the minimum and maximum number of clusters to 1 and 4 respectively. The optimum number of clusters representing potentially different acoustic groups from each ROI was defined by the Calinski-Harabasz Index (Caliński and Harabasz, 1974). The idea behind this K-means classification is to extract only one cluster having a single consistent biological structure from each ROI, thus forming one echo-type to ensure that the selected sample has a homogeneous frequency response (S2 Fig. 2a).


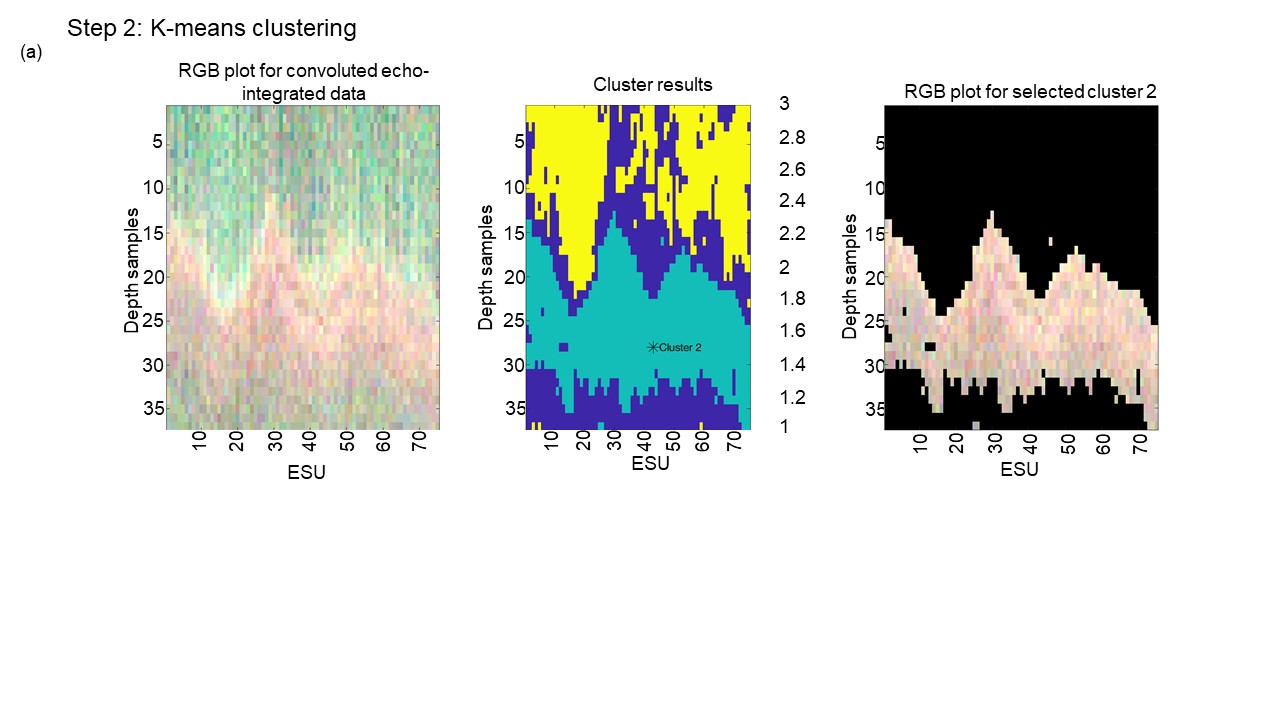


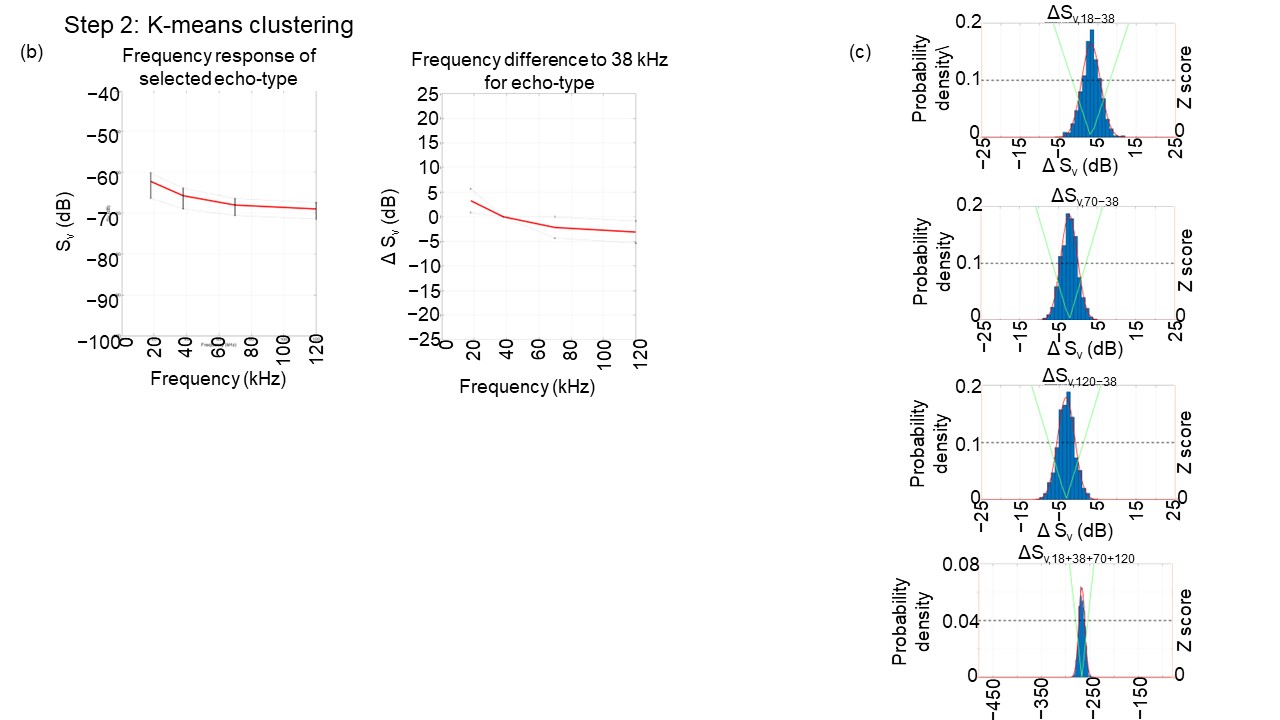


*Figure 2a. In the selected ROI, one cluster showing higher frequency response at 18 kHz was chosen following K-means clustering. (b) Mean frequency response curve (red) with standard deviations (black) of selected echo-type and the curve of frequency difference relative to 38 kHz for the chosen echo-type. (c) Histograms of ΔS_v,18−38_, ΔS_v,70−38_, ΔS_v,120−38_ for the echo-type is given with the corresponding probability density for a normal distribution.*

Histograms and probability density functions of the S_v_ differences (ΔS_v,18−38_, ΔS_v,70−38_, ΔS_v,120−38_) of each echo-type, were visualized. Only echo-types showing normal frequency distributions and limited variability in frequency responses were retained for the next step (S2 Fig 2b, c). At this step, we considered that the 215 selected echo-types include all relative frequency responses that are visible from the dataset at the four working frequencies.

**Step (3)** Three mean S_v_ differences (ΔS_v,18−38_, ΔS_v,70−38_, and ΔS_v,120−38_) were calculated from the 215 echo-types and classified using hierarchical cluster analysis in R software (v. 4.1.2) using the function “hclust” from the package stats (v. 3.6.2, R Core Team, 2021). The hierarchical dendrogram was constructed using Ward’s minimum variance method (Ward, 1963) and visualized with the “fviz_dend” function from the package factoextra (v. 1.0.7, Kassambara & Mundt, 2020) (S2 Fig. 3). The optimum number of echo-classes was determined using the “fviz-nbclust” function within the NbClust package (v. 3.0) that includes Euclidean distance and the ward.D2 method (Charrad *et al.*, 2014).


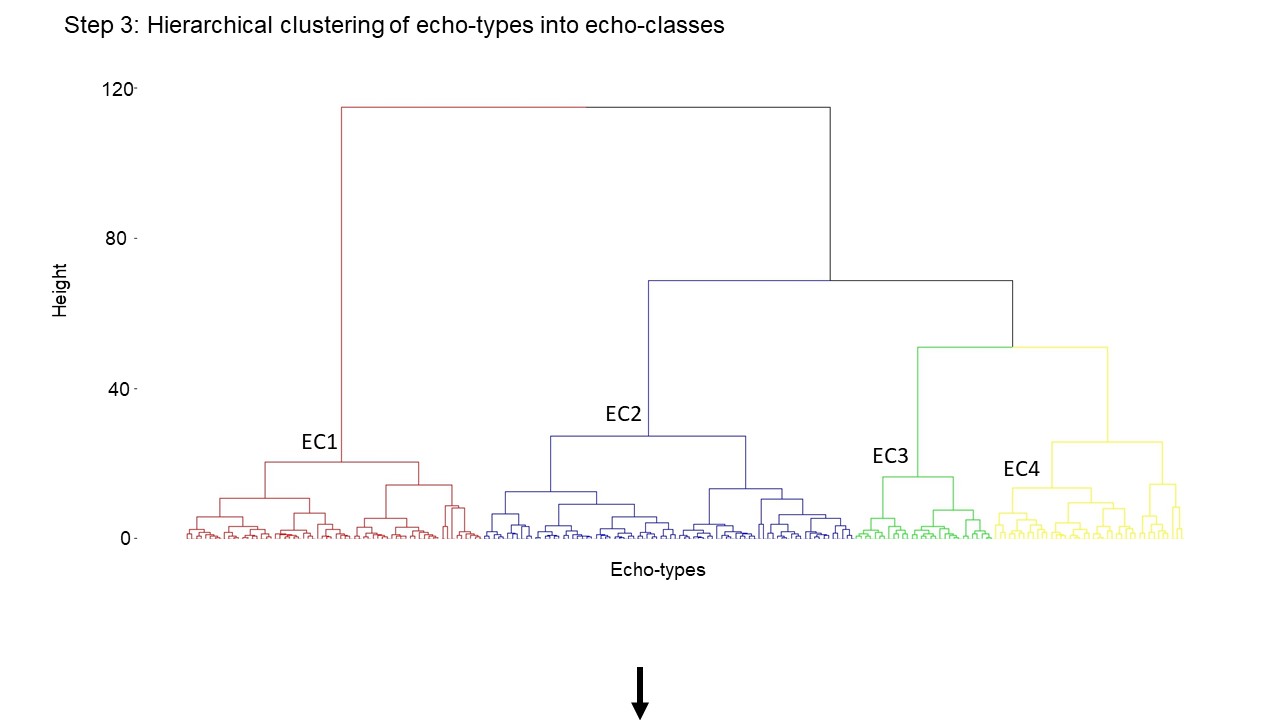


*Figure 3. A dendrogram using Ward’s clustering method showing the relative level of dissimilarity between the 215 echo-types. The echo-types were classified into 4 echo-classes (labelled EC1 through EC4).*

Cluster performance and echo-class classification error rates were determined from a random forest algorithm trained by the cluster-classified observations with ntree=500, using the R package randomForest (v. 4.7.1.1, Liaw and Wiener, 2002). Echo-types were partitioned in four clusters or echo-classes within the AC, C, and TZ. Echo-classes 1 to 4 had classification error rates of 0.04%, 0%, and 0.02%, implying that the hierarchical clustering and cluster-trained random forest robustly classified all echo-types correctly 99.4% of the time.

**Step (4)** The three mean dB differences characterizing the echo-classes constituted coordinates in a three-dimensional space where the orthogonal axes were defined by S_v_ differences between S_v18-38_ (x-axis), S_v70-38_ (y-axis) and S_v120-38_ (z-axis). The three-dimensional ordination plot was generated to visualize the four echo-classes along the S_v_ difference axes (S2 Fig. 4a). Data points representing the S_v_ differences of each echo-class were plotted around a centroid of an ellipsoid at mean ± 2 standard deviations (SD). Eigenvalues, rotation matrices and centroids represent the acoustic characteristics of each echo-class. The frequency responses of each echo-class were visualized (S2 Fig. 4b). The eigenvectors give the directions in which the echo-classes vary the most along each axis. The rotation matrix was defined as:

$R=(\frac{V1}{\left\| V1 \right\|}$ $\frac{V2}{\left\| V2 \right\|} \frac{V3}{\left\| V3 \right\|})$, Equation (1)

where $V1$, $V2$ and $V3$ are the eigenvectors relative to the eigenvalues λ1, λ2 and λ3 (λ1 > λ2 > λ3).


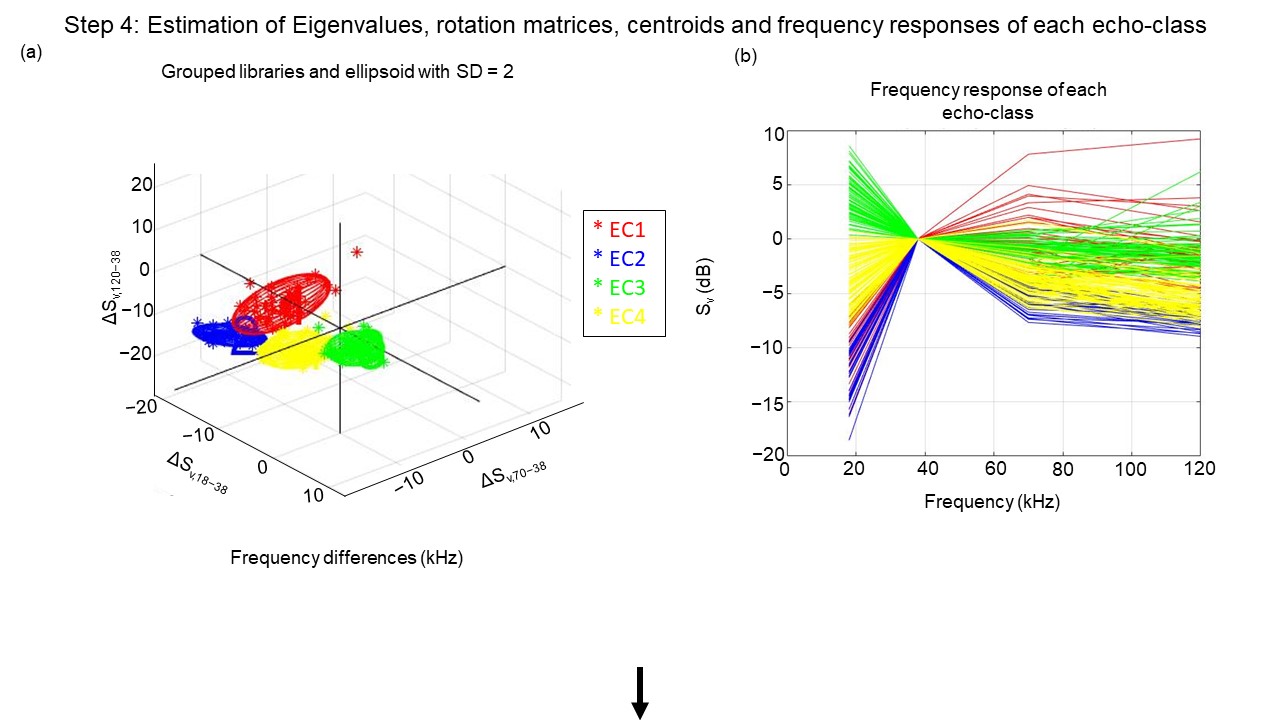


*Figure 4(a) Three-dimensional ordination plot representing the four echo-classes (EC1 through EC4) of the training dataset along S_v18-38_ (x-axis), S_v70-38_ (y-axis) and S_v120-38_ (z-axis). (b) Frequency response curves of each echo-class.*

**Step (5)** Each point in this three-dimensional space can be characterised by its distance from the centroid of each echo-class. This is what we call the Escore which is a sum of squared independent normal random variables following a chi-square distribution with three degrees of freedom. The Escore can be represented by the following equation:

${Escore}_{(i,k)}$= $\frac{x_{i}^{2}}{\lambda_{1(k)}}$ + $\frac{y_{i}^{2}}{\lambda_{2(k)}}+ \frac{z_{i}^{2}}{\lambda_{3(k)}}$ , Equation (2)

where $x_{i}$, $y_{i}$, and $z_{i}$ are the coordinates of the echo-integrated cell $i$ in the three-dimensional space defined by the ellipsoid $k$, and the origin is the arithmetic mean. For a given cell $i$, ${Escore}_{(i,k)}$ is calculated for each echo-class $k$. For that given cell, the higher the Escore, the lower is the similarity of the frequency response to the echo-class. In principle, the cell is assigned to the echo-class for which ${Escore}_{(i,k)}$ is the minimum.

In order to avoid classifying cells having frequency responses which are different from the defined echo-classes, a maximum threshold (Ellipsoid-threshold) is defined.

The Escore algorithm is:

If ${Escore}_{(i)}$ for all $k$ echo-classes >= Ellipsoid-threshold, cell $i$ is unclassified

If not, cell $i$ is assigned to echo-class $k$ according to smallest ${Escore}_{(i,k)}$

Sensitivity tests were conducted on the training dataset to evaluate the influence of the Ellipsoid-threshold value on the Escore algorithm (S2 Fig. 5). All cells of all echo-types were classified into echo-classes according to the conditions mentioned above (minimum Escore) by varying the Ellipsoid-threshold. This allows determination of the percentage of echo-integrated cells of each echo-class that were well classified, mis-classified and not classified. An Ellipsoid-threshold at a fixed value of 25 was chosen to optimize the percentage echo-integration cells and s_A_ well classified and limit those which were mis-classified and decrease those which were not classified for each echo-class.


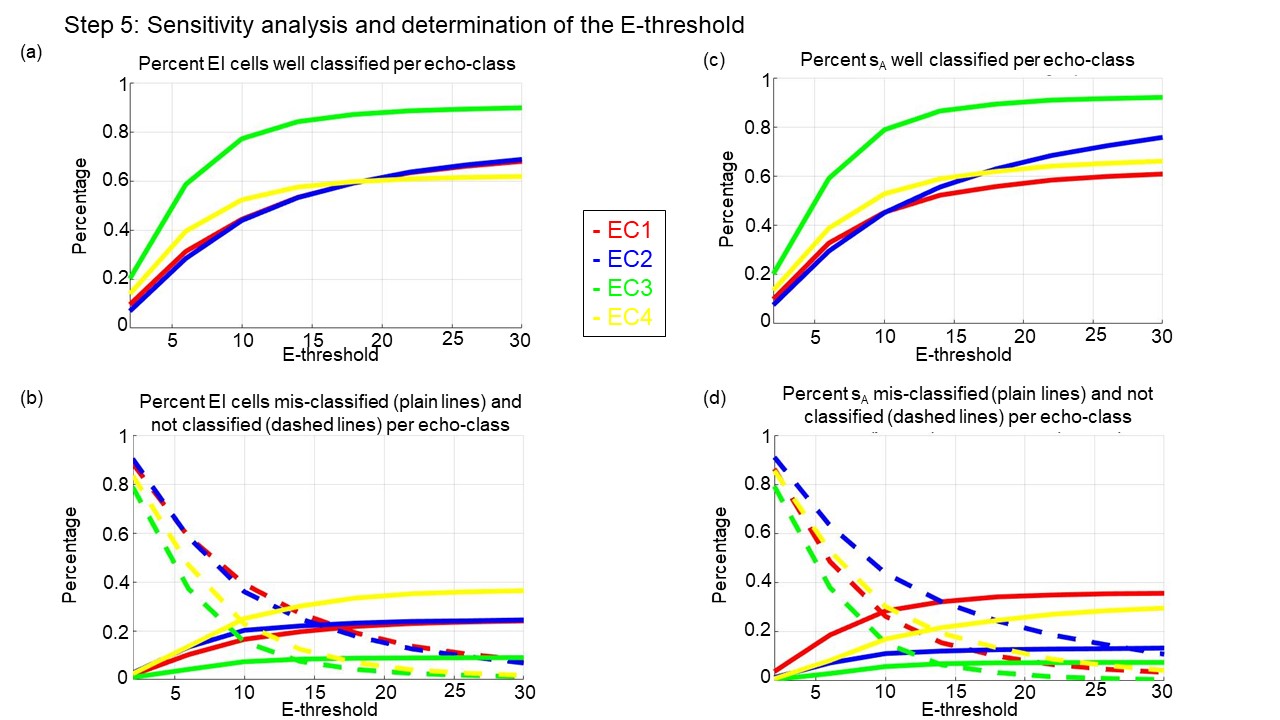


*Figure 5. Graphs of the percentage echo-integrated (EI) cells (a) well classified, (b) mis-classified (plain lines) and not classified (dashed lines) per echo-class. Graphs of the percentage s_A_ (c) well classified, (d) mis-classified (plain lines) and not classified (dashed lines) per echo-class.*

**Step (6)** The Escore algorithm (with Ellipsoid-threshold = 25) was run on the whole acoustic dataset to classify each echo-integrated cell into one of the four echo-classes (S2 Fig. 6). Echograms were generated for each echo-class.


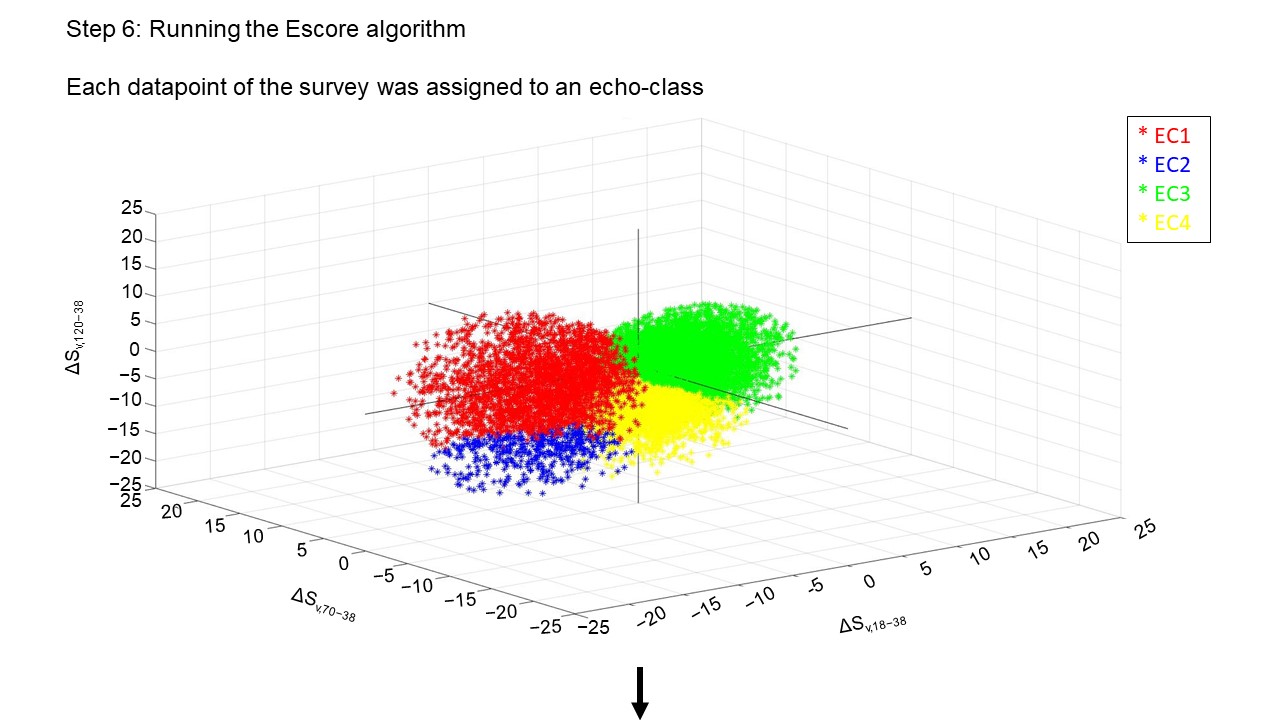


*Figure 6. Three-dimensional ordination plot representing the four echo-classes (EC1 through EC4) of the whole acoustic dataset along S_v18-38_ (x-axis), S_v70-38_ (y-axis) and S_v120-38_ (z-axis).*

**References**

Annasawmy P, Ternon J-F, Cotel P, Cherel Y, Romanov EV, Roudaut G, Lebourges-Dhaussy A. Ménard F, Marsac F. Micronekton distributions and assemblages at two shallow seamounts of the south-western Indian Ocean: Insights from acoustics and mesopelagic trawl data. Progress in Oceanography. 2019;178: 102161. https://doi.org/10.1016/j.pocean.2019.102161

Burgos JM, Horne JK. Characterization and classification of acoustically detected fish spatial distributions. ICES Journal of Marine Science. 2008;65:1235–1247.

Caliński T, Harabasz J. A dendrite method for cluster analysis. Communications in Statistics. 1974;3(1).

Charrad M, Ghazzali N, Boiteau V, Niknafs A. NbClust: An R package for determining the relevant number of clusters in a data set. J Stat Softw. 2014;61:1–36

Fernandes PG. Classification trees for species identification of fish-school echotraces. ICES Journal of Marine Science. 2009;66:1073–1080. https://doi.org/10.1093/icesjms/fsp060

Kassambara A, Mundt F. factoextra: extract and visualize the results of multivariate data analyses (version 1.0.7). 2020. https:// CRAN.R-project.org/package=factoextra

Liaw A, Wiener M. Classification and Regression by randomForest. R News. 2002;2(3): 18-22. <https://CRAN.R-project.org/doc/Rnews/>

R Core Team. R: A language and environment for statistical computing. 2021. R Foundation for Statistical Computing, Vienna, Austria. https://www.R-project.org/.

Ward JH. Hierarchical grouping to optimize an objective function. J Am Stat Assoc. 1963;58:236–244
