# Supplementary material for: Impact of an eddy dipole of the Mozambique channel on mesopelagic organisms, highlighted by multifrequency backscatter classification
Source: PLoS One. 2024 Sep 11;19(9):e0309840. doi: 10.1371/journal.pone.0309840 (PMC12139656; doi:10.1371/journal.pone.0309840)
Supplement: S3 File — (DOCX) [file pone.0309840.s003.docx]

**S3** **Description of theoretical scattering models**

The distorted-wave born approximation (DWBA) model was used for all frequencies and angles of orientation to describe groups of fluid-like zooplankton – small euphausiids based on a uniformly bent cylinder and copepods on a prolate spheroid (Stanton and Chu, 2000), high pass fluid-sphere applicable only to shelled gastropods, decapod shrimp and salp (randomly-oriented fluid, bent cylinder; DWBA of Stanton *et al.* (1993) with the constants specified in the equation of Stanton *et al.* 1994), and siphonophore (single gas inclusion) of varying sizes (high-pass model initially defined in Stanton *et al.* (1989)). A hybrid scattering model from Barbin *et al.* 2024 describing gas-bearing organisms, i.e., fish with swimbladders and siphonophore with pneumatophore, was used in this study.

The model parameters and full model descriptions used in this study had previously been validated through comparison with laboratory or *in situ* measurements (Lavery *et al.*, 2007) and are given in the tables below.

| **Organism (Scattering model)** | **Estimated spherical radius (a, mm)** | **Density contrast (g)** | **Sound speed contrast (h)** | **Orientation (Mean, STD)** | **Length to width ratio (L/2a)** | **References** |
| --- | --- | --- | --- | --- | --- | --- |
| Large euphausiids (DWBA) | 3: 16 | 5.485*L/10^4^ +1.002 | 5.942*L/10^4^ +1.004 | (20°, 20°) | 5.25 | Lavery *et al*., 2007 for L > 25 mm |
| Small euphausiids (DWBA) | 0.5: 2.3 | 1.016 | 1.019 | (20°, 20°) | 5.25 | Lavery *et al*., 2007 for L < 25 mm |
| Gas bubble – spherical | 0.05: 10 | 0.0024 | 0.22 | (0°, 0°) | 1 | Stanton *et al.*, 1998 |
| Gas bubble – ellipsoid | 0.05: 10 | 0.0024 | 0.22 | (0°, 30°) | 1.5 | Stanton *et al.*, 1998 |
| Copepod (DWBA) | 0.05: 3 | 1.12 | 1.09 | (0°, 30°) | 2.55 | Stanton and Chu, 2000 |

| **Scattering model** | **Estimated spherical radius (a, mm)** | **BetaD (L/2a)** | **Reflection coefficient** | **References** |
| --- | --- | --- | --- | --- |
| Fluid, bent cylinder - salp | 0.5: 5 | 1.7 | 0.0041 | Stanton *et al.,* 1994 |
| Fluid, bent cylinder - shrimp | 0.5: 5 | 8 | 0.058 | Stanton *et al.,* 1994 |
| High pass fluid-sphere - gastropod | 0.5: 2 | 1 | 0.5 | Stanton *et al.,* 1994 |

| **Scattering model** | **Organism size (mm)** | **Percentage of swimbladder in fish body (%)** | **L/2a fish body ratio** | **L/2a gaseous inclusion ratio** | **Gamma** | **Incidence (°)** | **References** |
| --- | --- | --- | --- | --- | --- | --- | --- |
| Hybrid model – value for swimbladdered fish | 5: 185 | 2.5 | 5 | 1.5 | 1.4 | 0 | Barbin *et al.*, 2024 |

| **Scattering model** | **Organism size (mm)** | **L/2a** | **Gamma** | **References** |
| --- | --- | --- | --- | --- |
| Hybrid model – value for pneumatophore | 0.1:1.5 | 1.35 | 1.4 | Barbin *et al.*, 2024 |

The models were run using the temperature, salinity and speed of sound in water at 25, and 200 m depths. The following environmental parameters were used to parametrize the scattering models:

| Depth (m) | Temperature (°C) | Salinity | Speed of sound in water (m^2^ s^-2^) using Mackenzie equation |
| --- | --- | --- | --- |
| 25 | 28 | 35.2 | 1541.75 |
| 200 | 16 | 35.4 | 1513.55 |

Scattering models were classified into echo-classes 1 to 4 (in red, blue, green, and yellow, respectively) as shown in the figure below:


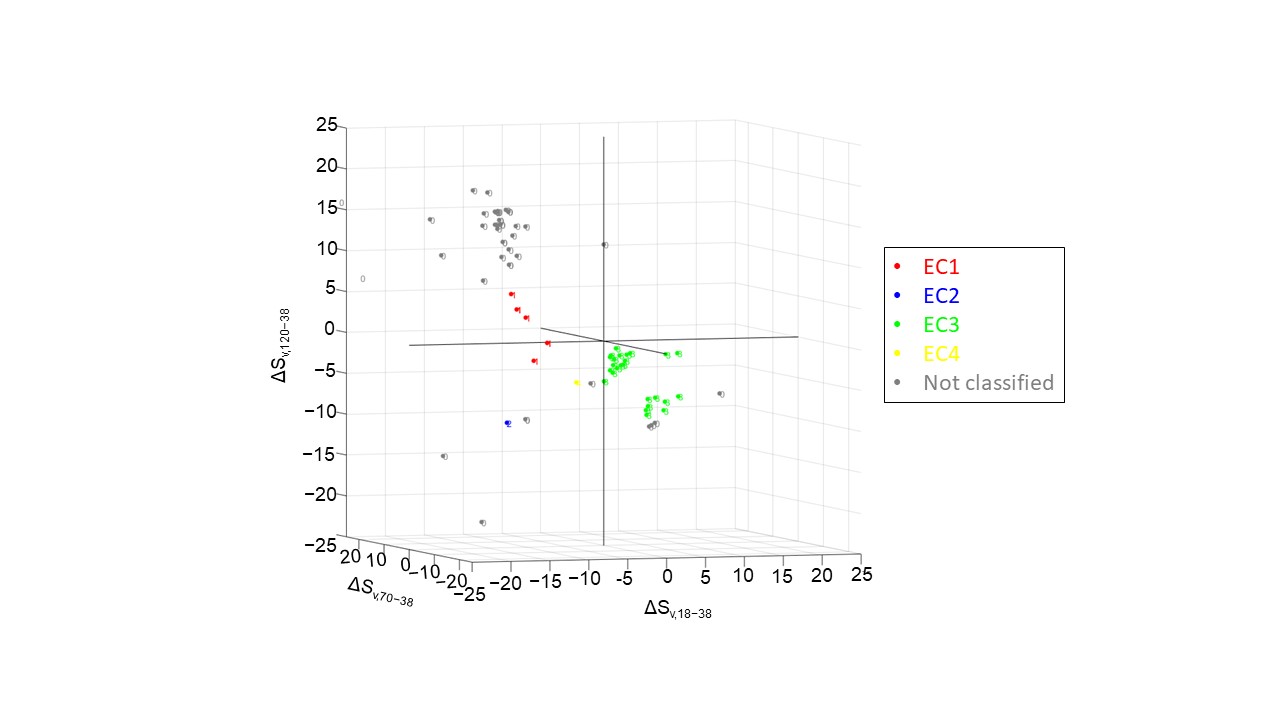


The following models (grey in the above figure) were not classified into any echo-class:

| **Scattering models not classified into any echo-class** | **Estimated spherical radius (a, mm)** |
| --- | --- |
| Hybrid Fish | 5 |
| Hybrid Siphonophore | 0.1 |
| Copepod (DWBA) | 0.05 |
| Copepod (DWBA) | 0.08 |
| Copepod (DWBA) | 0.12 |
| Copepod (DWBA) | 0.2 |
| Copepod (DWBA) | 0.31 |
| Copepod (DWBA) | 0.49 |
| Copepod (DWBA) | 0.77 |
| High pass fluid-sphere - gastropod | 0.5 |
| High pass fluid-sphere - gastropod | 0.71 |
| High pass fluid-sphere - gastropod | 1 |
| High pass fluid-sphere - gastropod | 1.41 |
| High pass fluid-sphere - gastropod | 2 |
| Fluid, bent cylinder - salp | 0.5 |
| Fluid, bent cylinder - salp | 0.65 |
| Fluid, bent cylinder - salp | 0.83 |
| Fluid, bent cylinder - salp | 1.08 |
| Fluid, bent cylinder - salp | 1.39 |
| Fluid, bent cylinder - salp | 1.8 |
| Fluid, bent cylinder - salp | 2.32 |
| Fluid, bent cylinder - salp | 3.87 |
| Fluid, bent cylinder - shrimp | 0.5 |
| Fluid, bent cylinder - shrimp | 0.65 |
| Fluid, bent cylinder - shrimp | 0.83 |
| Fluid, bent cylinder - shrimp | 1.08 |
| Fluid, bent cylinder - shrimp | 1.39 |
| Fluid, bent cylinder - shrimp | 1.8 |
| Fluid, bent cylinder - shrimp | 2.32 |
| Fluid, bent cylinder - shrimp | 3.87 |
| Gas bubble - ellipsoid | 0.05 |
| Gas bubble - ellipsoid | 0.09 |
| Gas bubble - ellipsoid | 0.16 |
| Gas bubble - ellipsoid | 0.29 |
| Gas bubble - ellipsoid | 5.55 |
| Gas bubble - spherical | 0.05 |
| Gas bubble - spherical | 0.09 |
| Gas bubble - spherical | 0.29 |
| Gas bubble - spherical | 0.53 |
| Gas bubble - spherical | 3.08 |
| Large euphausiids (DWBA) | 6.9 |
| Large euphausiids (DWBA) | 16 |
| Small euphausiids (DWBA) | 0.5 |
| Small euphausiids (DWBA) | 0.73 |
| Small euphausiids (DWBA) | 1.07 |
| Small euphausiids (DWBA) | 1.57 |
| Small euphausiids (DWBA) | 2.3 |
