# Supplementary material for: Impact of an eddy dipole of the Mozambique channel on mesopelagic organisms, highlighted by multifrequency backscatter classification
Source: PLoS One. 2024 Sep 11;19(9):e0309840. doi: 10.1371/journal.pone.0309840 (PMC12139656; doi:10.1371/journal.pone.0309840)
Supplement: S4 File — (DOCX) [file pone.0309840.s004.docx]

**S4 Acoustic Modelling – GAMM**

GAMMs were used to investigate the candidate covariates influencing the acoustic densities of each echo-class. A restricted maximum likelihood approach and penalized thin plate regression splines were used on all smooth terms with a conservative value of k < 10 to constrain overfitting (Marra and Wood, 2011). The model fit was iteratively checked by varying the k value and calculating the k-index and performing a diagnostic of the GAMM to assess whether the basis dimension choices were adequate. An index value close to 1 or greater indicates an adequate basis dimension for the smoothing functions. Prior to running the GAMMs, collinearity among the environmental variables were evaluated by plotting scatterplot matrices of each variable as a function of day and night using the R package GGally (v. 2.1.2; Schloerke *et al.*, 2021), and investigating pairwise relationships between variables using Pearson’s correlation coefficient (See S5). Data distributions of each variable were visually assessed and the appropriate data transformation was selected (square-root or log transformation) before running the GAMMs using a Gaussian family with an identity link function. Assumptions of variance homogeneity and normality were visually assessed using residual plots. Deviance explained (analogous to variance explained in a linear regression) and adjusted r^2^ were used as indicators of model performance.

Candidate environmental variables

The candidate covariates considered were the mean eddy kinetic energy (EKE) between 24-100 m, 100-200 m, 200-248 m derived from the S-ADCP, and fluorescence (Fluo), temperature (Temp) and salinity (Salt) between 15-100 m, 100-200 m, and 200-250 m measured by the MVP. Binomial factors included “day” and “night”. Based on density plots and histograms, the mean EKE and s_A_ were square-root transformed and MVP data were log transformed prior to GAMM fitting whenever the distributions deviated from normality so as to downweigh extreme values.

Model selection

A full candidate model specification was first tested for each echo-class:

s_A_15-100 + s_A_100-200 + s_A_200-250 ~ s(EKE24-100, k =3) + s(EKE100_200, k=3) +s(EKE200_248, k=3) +s(Fluo15_100, k=3) +s(Fluo100_200, k=3)+ s(Fluo200_250, k=3)+s(Salt15_100, k=3)+ s(Salt100_200, k=5)+ s(Salt200_250, k=3)+ s(Temp15_100, k=3)+ s(Temp100_200, k=3)+ s(Temp200_250, k=3)+ Time_of_day, method="REML", na.action="na.fail", correlation=corAR1(form= ~Interval|Cruise_ID)

In the fitted GAMMs, “Interval” is a numeric value that sequentially identifies each 1 km distance (1, 2, 3, … i), and cruise is a unique cruise leg identifier, i.e., autocorrelation structure is fitted sequentially. “REML” is the restricted maximum likelihood approach and k is the number of basis functions.

Where collinearity between environmental variables of each echo-class dataset was identified from the scatterplot matrices at a cut off value of 0.8 (similar to Boswell *et al.* 2020), the correlations between these collinear variables and s_A_ were assessed. Of the two environmental variables that are highly collinear, the one which show greater correlation with s_A_ was retained in the final models and the other variable was eliminated to reduce the possibility of Type II errors (S5). Final models were hence tested for each echo-class with selected explanatory variables.

Echo-class 1:

s_A_15_100 + s_A_100_200 + s_A_200_250 ~ s(EKE200_248, k=3) + s(EKE24_100, k=3) + s(Fluo15_100, k=3) + s(Fluo100_200, k=3)+ s(Fluo200_250, k=3) + s(Salt15_100, k=3)+ s(Salt200_250, k=3) + s(Temp100_200, k=3)+ s(Temp200_250, k=3) + Time_of_day, method="REML", na.action="na.fail", correlation=corAR1(form= ~Interval|Cruise_ID)

Echo-class 2:

s_A_15_100 + s_A_100_200 + s_A_200_250 ~ s(EKE24_100) k=3) + s(Fluo15_100, k=3) + s(Fluo100_200, k=3) + s(Salt15_100, k=3) + s(Salt200_250, k=3) + s(Temp15_100, k=3), method="REML", na.action="na.fail", correlation=corAR1(form= ~Interval|Cruise_ID)

Echo-class 3:

s_A_15_100 + s_A_100_200 + s_A_200_250 ~ s(EKE24_100, k=10) + s(EKE100_200, k=10) + s(EKE200_248, k=10) + s(Fluo100_200, k=10)+ s(Fluo200_250, k=3) + s(Salt15_100, k=10) + s(Salt100_200, k=3) + s(Salt200_250, k=10) + s(Temp15_100, k=5) + s(Temp100_200, k=5) + Time_of_day, method="REML", na.action="na.fail", correlation=corAR1(form= ~Interval|Cruise_ID)

Echo-class 4:

s_A_15_100 + s_A_100_200 + s_A_200_250 ~ s(EKE24_100, k=5) + s(EKE100_200, k=5) +s(EKE200_248, k=3) + s(Fluo100_200, k=3)+ s(Fluo200_250, k=3) + s(Salt15_100, k=3) + s(Salt100_200, k=5) + s(Salt200_250, k=5) + s(Temp15_100, k=10) + s(Temp200_250, k=3) + Time_of_day, method="REML", na.action="na.fail", correlation=corAR1(form= ~Interval|Cruise_ID)

**References**

Boswell KM, D’Elia M, Johnston MW, Mohan JA, Warren JD, Wells RJD, Sutton TT. Oceanographic Structure and Light Levels Drive Patterns of Sound Scattering Layers in a Low-Latitude Oceanic System. Frontiers in Marine Science. 2020;7: 51. https://doi.org/10.3389/fmars.2020.00051

Schloerke B, Cook D, Larmarange J, Briatte F, Marbach M, Thoen E, Elberg A, Crowley J. GGally: extension to 'ggplot2'. R package version 2.1.2. 2021. https://CRAN.R-project.org/package= GGally
