# Supplementary material for: Impact of an eddy dipole of the Mozambique channel on mesopelagic organisms, highlighted by multifrequency backscatter classification
Source: PLoS One. 2024 Sep 11;19(9):e0309840. doi: 10.1371/journal.pone.0309840 (PMC12139656; doi:10.1371/journal.pone.0309840)
Supplement: S5 File — (DOCX) [file pone.0309840.s005.docx]

**S5 Testing for collinearity between environmental covariates**

Echo-class 1


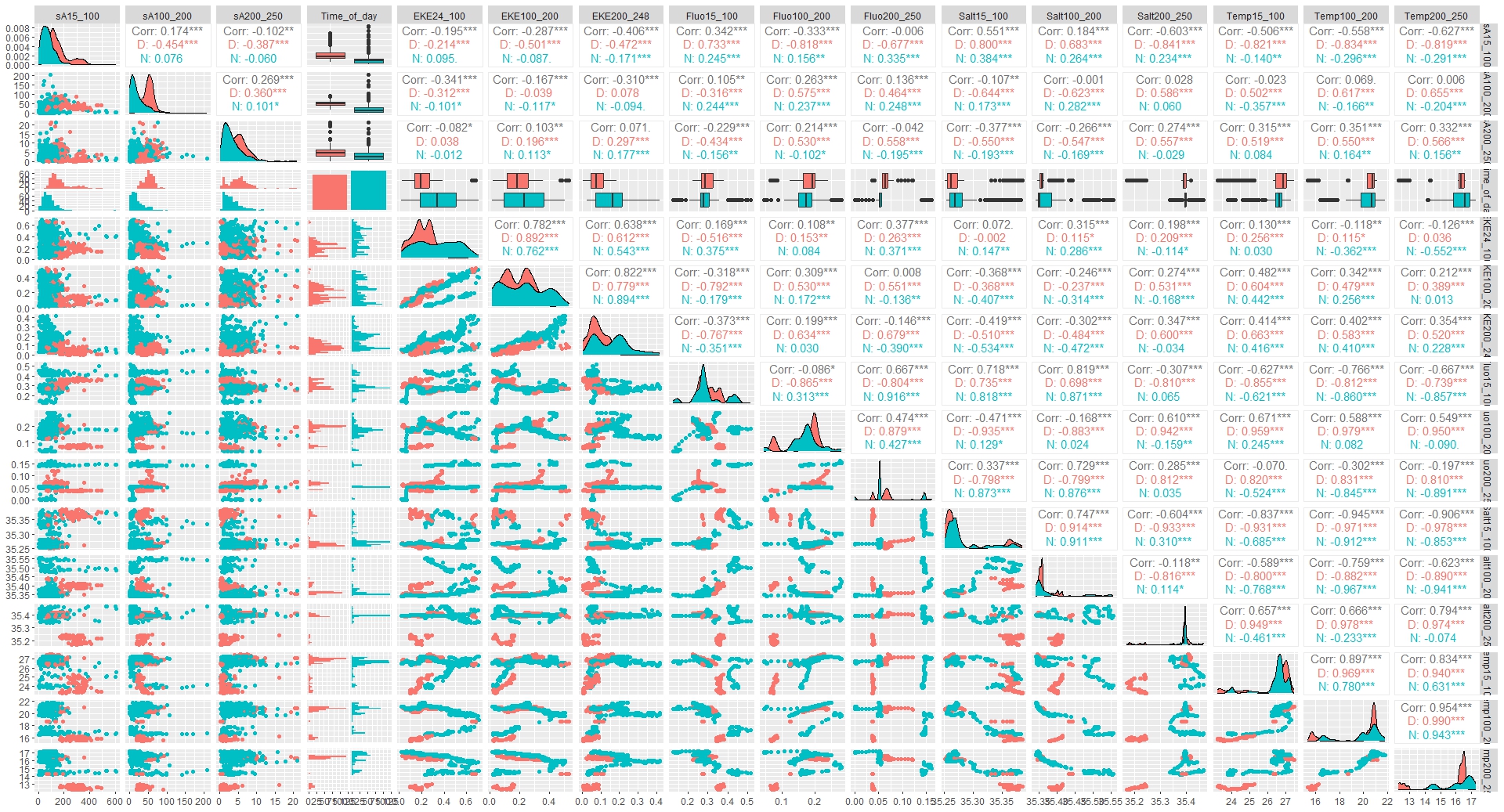


Scatterplot matrix of response variable, s_A_, and candidate environmental variables during day time (pink color) and night time (blue color) to assess for collinearity. Since several candidate covariates showed correlation coefficients (Corr) > 0.8, out of the two collinear environmental variables, the one which is the most highly correlated to s_A_ was retained in the final models, and the others dropped. Data distributions are shown along the lower diagonal.

Echo-class 2


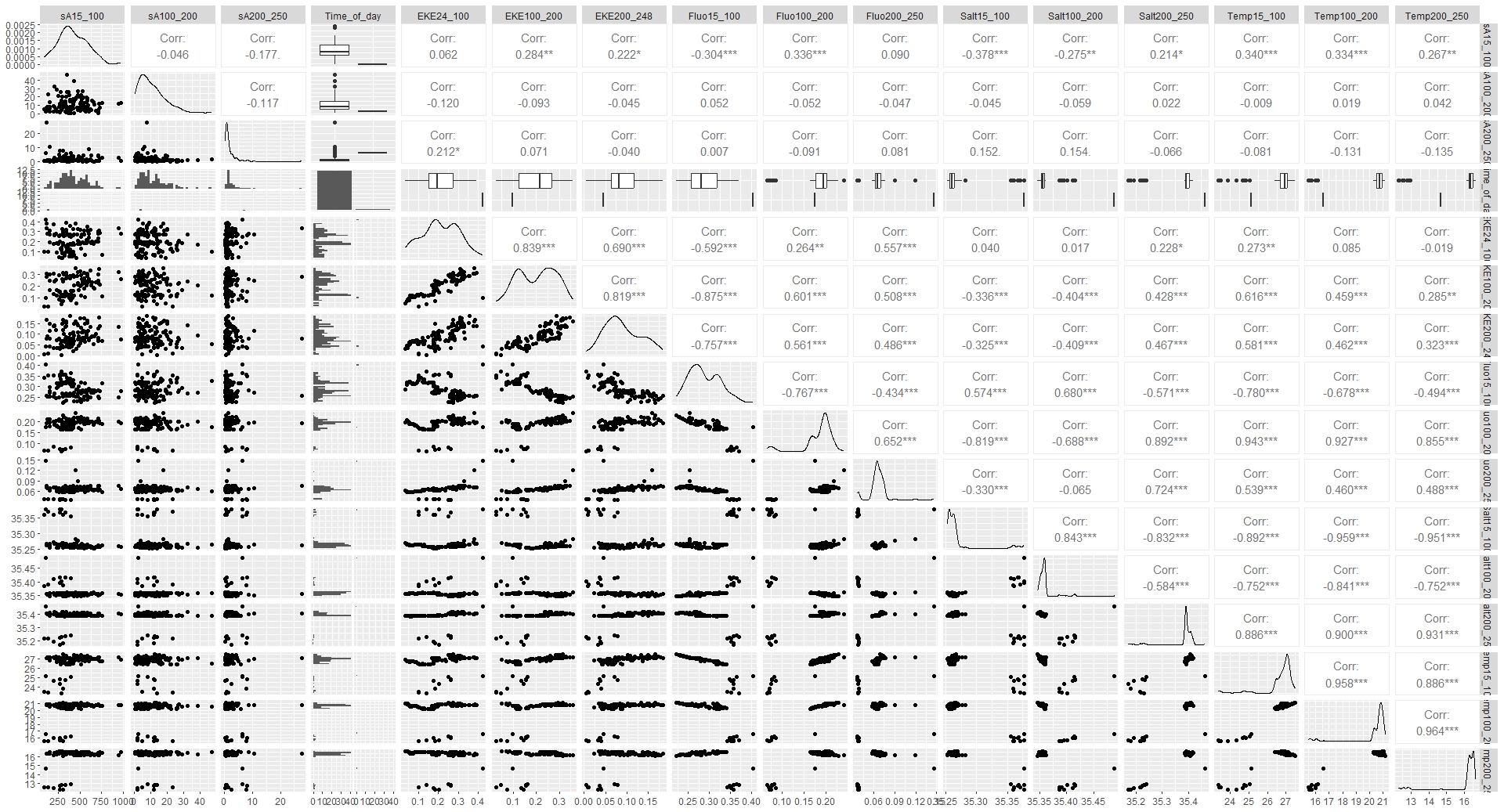


Scatterplot matrix of response variable, s_A_, and candidate environmental variables to assess for collinearity. Since several candidate covariates showed correlation coefficients (Corr) > 0.8, out of the two collinear environmental variables, the one which is the most highly correlated to s_A_ was retained in the final models, and the others dropped. Data distributions are shown along the lower diagonal.

Echo-class 3


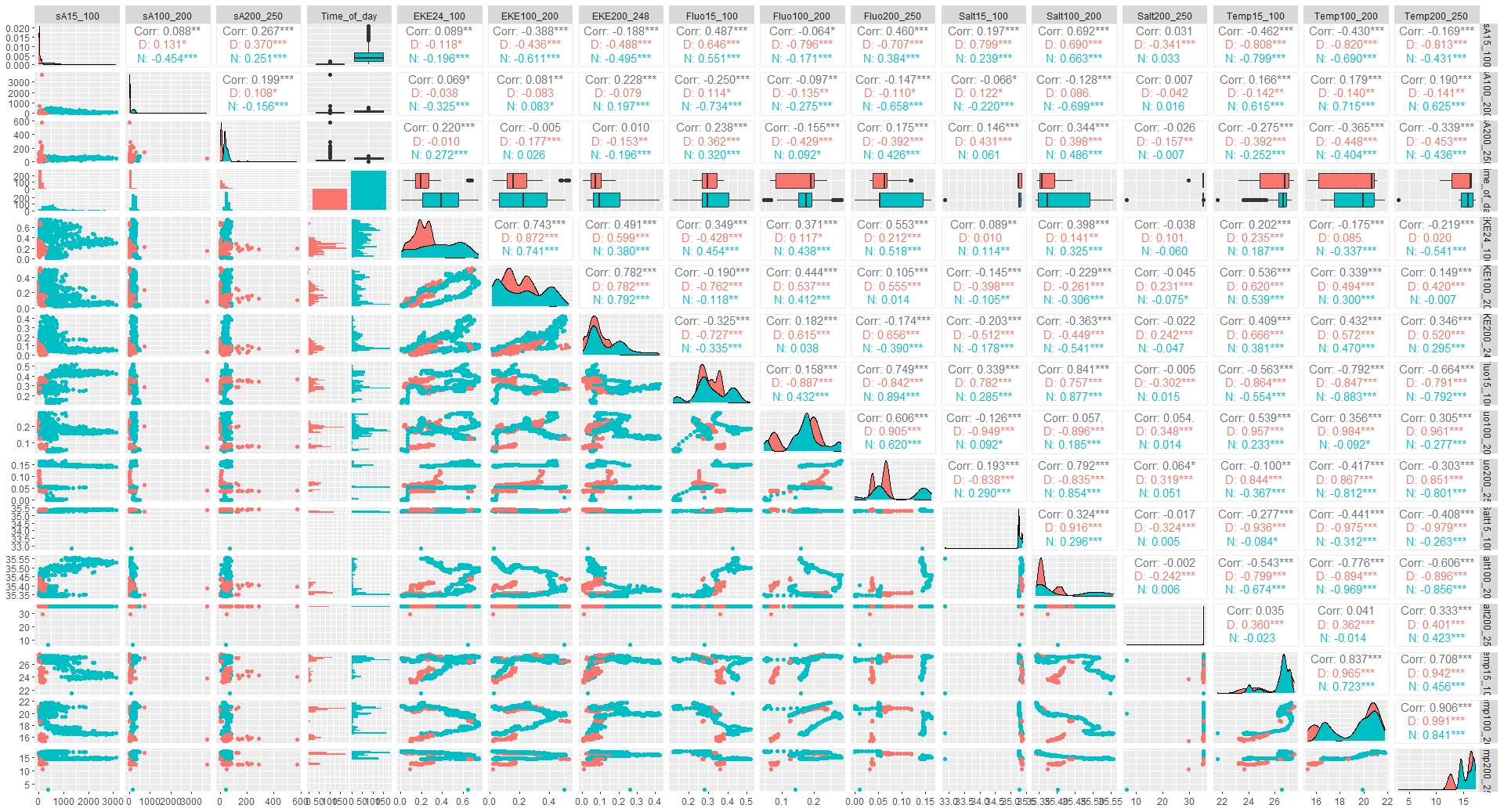


Scatterplot matrix of response variable, s_A_, and candidate environmental variables during day time (pink color) and night time (blue color) to assess for collinearity. Since several candidate covariates showed correlation coefficients (Corr) > 0.8, out of the two collinear environmental variables, the one which is the most highly correlated to s_A_ was retained in the final models, and the others dropped. Data distributions are shown along the lower diagonal.

Echo-class 4


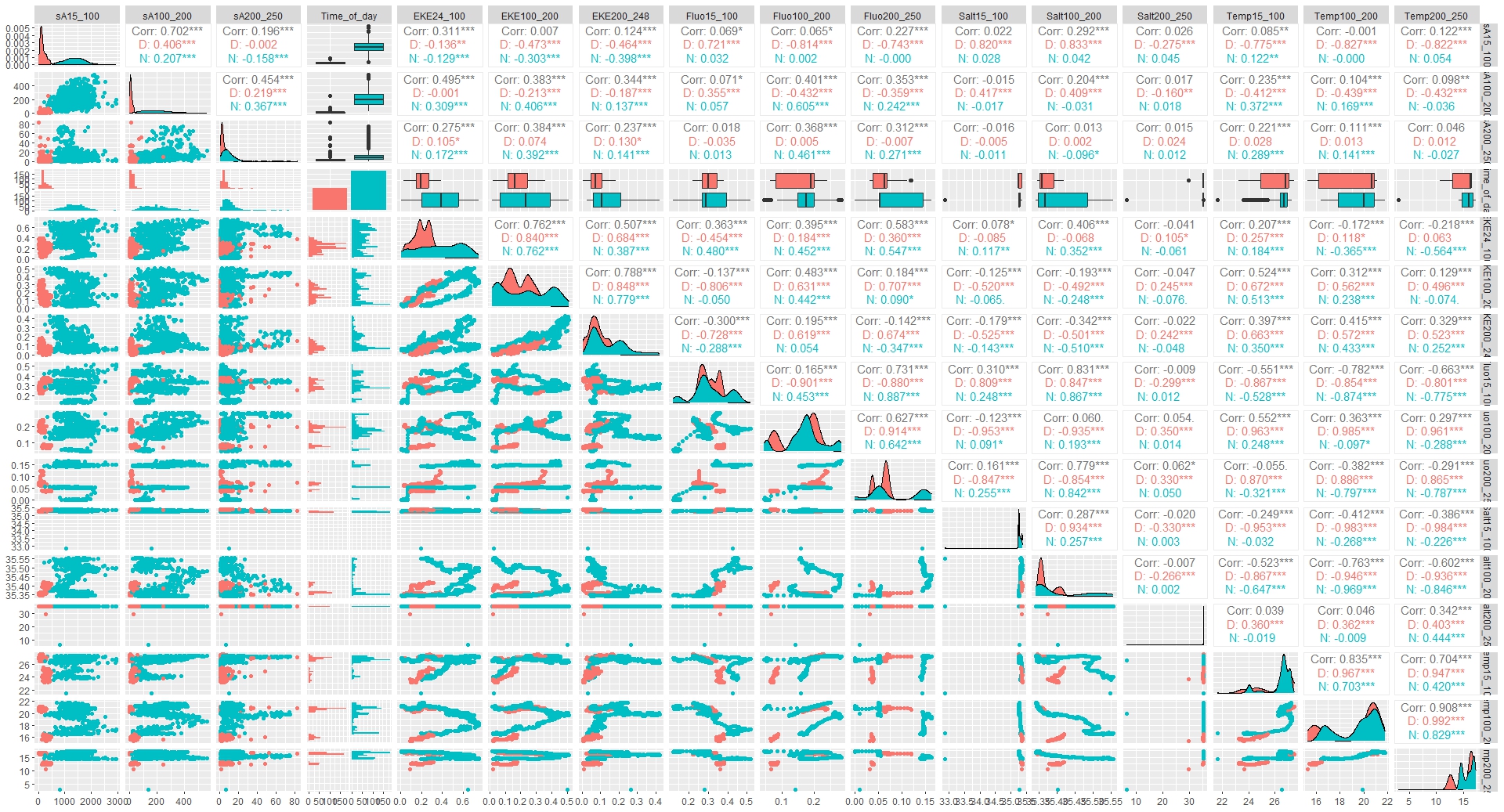


Scatterplot matrix of response variable, s_A_, and candidate environmental variables during day time (pink color) and night time (blue color) to assess for collinearity. Since several candidate covariates showed correlation coefficients (Corr) > 0.8, out of the two collinear environmental variables, the one which is the most highly correlated to s_A_ was retained in the final models, and the others dropped. Data distributions are shown along the lower diagonal.
